# LAIOR: a hyperbolic neural ODE variational framework for interpretable single-cell manifold learning and trajectory inference

**DOI:** 10.3389/fgene.2026.1838613

**Published:** 2026-06-08

**Authors:** Zeyu Fu, Jiawei Fu, Keyang Zhang, Tianfei Ran, Chunlin Chen

**Affiliations:** 1 State Key Laboratory of Trauma and Chemical Poisoning, Institute of Combined Injury, Chongqing Engineering Research Center for Nanomedicine, College of Preventive Medicine, Army Medical University, Chongqing, China; 2 Department of Orthopedics, Xinqiao Hospital, Army Medical University, Chongqing, China; 3 School of Medicine, Sun Yat-sen University, Shenzhen, China; 4 Department of Rehabilitation Medicine, The First Affiliated Hospital, Sun Yat-sen University, Guangzhou, China

**Keywords:** benchmarking, dynamics modeling, hyperbolic geometry, information bottleneck, manifold learning, single-cell

## Abstract

Single-cell omics data are high-dimensional, sparse, and noisy, and learning embeddings that simultaneously preserve local cell-state structure, global hierarchy, and smooth developmental trajectories remains an open problem. Existing approaches typically achieve only one of these goals: classical methods emphasize either local neighborhoods or global variance; deep generative models cluster cell types well but often fracture trajectory continuity; and hyperbolic embeddings capture hierarchy but are numerically fragile in practice. We present LAIOR (Lorentz attentive interpretable ordinary differential equation (ODE)-regularized variational autoencoder (VAE)), a unified variational framework that combines three complementary inductive biases in a single forward pass: (i) *Lorentz geometric regularization* encourages tree-like latent hierarchy while remaining numerically stable *via* tangent-space clamping and exponential-map gating; (ii) a *dual-path information bottleneck* captures coordinated biological programs rather than forcing latent independence; and (iii) *neural ordinary differential equation (ODE) regularization* stabilizes latent trajectories through explicit learned dynamics. Across 118 single-cell datasets (53 scRNA-seq and 65 scATAC-seq) benchmarked against 23 baseline methods on 22 complementary metrics, LAIOR improves manifold continuity, trajectory coherence, and embedding fidelity while retaining competitive clustering performance. Ablation and sensitivity analyses show that ODE regularization stabilizes geometric learning and dampens hyperparameter sensitivity. Architecture interpretation experiments on two well-characterized reference systems (human bone marrow and mouse pancreatic endocrinogenesis) demonstrate that LAIOR’s encoder and decoder pathways decompose cellular variation into mutually exclusive, biologically coherent latent modules, and biological validation experiments on two previously unseen hematopoietic perturbation cohorts (*Dapp1* knockout and chemotherapy-induced bone marrow failure) show that the same interpretability contract transfers to perturbed biology. LAIOR generalizes across RNA and chromatin accessibility modalities without architectural changes. Head-to-head comparisons against both dynamical baselines (scTour) and foundation models (scGPT, scFoundation) confirm that explicit geometric and dynamical inductive biases recover trajectory structure that large-scale pretraining alone does not. Together, these results establish LAIOR as a practical, interpretable framework for single-cell manifold and trajectory analysis.

## 1 Introduction

Single-cell omics technologies have transformed the study of cellular heterogeneity by profiling molecular states at single-cell resolution (Tang et al., 2009; Kolodziejczyk et al., 2015). Single-cell RNA sequencing (scRNA-seq) captures transcriptomic landscapes, while single-cell assay for transposase-accessible chromatin (scATAC-seq) maps regulatory architecture (Bu et al., 2015; Cusanovich et al., 2015). However, the high dimensionality, sparsity, and technical noise inherent in these data present fundamental challenges for computational analysis (Brennecke et al., 2013; Grün et al., 2014). A central objective is to learn low-dimensional latent representations that preserve both local neighborhood structure, which is critical for cell-type identification, and global topology, which is essential for understanding developmental hierarchies and differentiation trajectories (Haghverdi et al., 2016; Trapnell et al., 2014).

### 1.1 The local–global trade-off in single-cell embeddings

Classical dimensionality reduction methods prioritize either local or global structure, but rarely both. Principal component analysis (PCA) captures global variance but distorts local neighborhoods due to linearity constraints (Jolliffe and Cadima, 2016). Non-linear methods such as t-SNE (van der Maaten and Hinton, 2008) and UMAP (McInnes et al., 2018) excel at preserving local clusters but systematically compress global distances, obscuring hierarchical relationships (Kobak and Berens, 2019; Becht et al., 2019). This trade-off propagates into downstream analyses: trajectory-inference algorithms trained on distorted embeddings may misidentify differentiation pathways, while batch correction methods can inadvertently erase biological gradients (Saelens et al., 2019; Tran et al., 2020).

Recent deep generative models such as scVI (Lopez et al., 2018) and scGNN (Wang et al., 2021) demonstrate robust performance in discrete cell-type clustering by modeling count distributions and incorporating graph regularization. However, evaluations reveal persistent deficiencies in global topology preservation: distance correlations between high-dimensional and latent spaces remain weak, and trajectory directionality exhibits inconsistency (Saelens et al., 2019; Luecken et al., 2022). Factor-based VAE variants ( β -VAE (Higgins et al., 2017), InfoVAE (Zhao et al., 2019)) prioritize statistical independence for interpretability, but axis-aligned factorization conflicts with the intrinsic geometry of cellular differentiation, where biological processes exhibit coordinated, hierarchical organization (Buettner et al., 2015).

### 1.2 Hyperbolic geometry and neural ordinary differential equation (ODE) dynamics

Biological systems frequently exhibit tree-like hierarchies: hematopoietic stem cells differentiate into progressively specialized lineages, developmental programs branch into distinct fates, and immune responses generate clonal expansions (Laurenti and Göttgens, 2018; Wagner and Klein, 2020). Hyperbolic space with constant negative curvature enables exponentially more efficient tree embeddings, requiring O(log⁡n) dimensions versus O(n) in Euclidean space (Sarkar, 2011; Krioukov et al., 2010). This has motivated the use of hyperbolic methods, including Poincaré maps (Klimovskaia et al., 2020), probabilistic geometric models (Poincaré Gaussian mixture, or PGM; Mathieu et al., 2019), and hyperbolic graph networks (Nagano et al., 2019; Tian T. et al., 2023).

Despite theoretical advantages, existing hyperbolic approaches face critical limitations. Fully hyperbolic latent variable models require all operations within hyperbolic manifolds, introducing numerical instabilities from arccosh singularities and gradient vanishing (Ganea et al., 2018; Skopek et al., 2020). Pure geometric embeddings lack mechanisms to enforce temporal consistency, leaving them unstable under biological noise (Street et al., 2018). Most methods optimize for either hierarchical structure or local clustering but do not systematically integrate both objectives (Wolf et al., 2019).

Cellular differentiation unfolds as continuous dynamical processes governed by regulatory programs (Weinreb et al., 2018; La Manno et al., 2018). Neural ordinary differential equations (ODEs) provide a natural framework by parameterizing latent trajectories as solutions to $\frac{dz}{dt}=f_{\theta }\left(z,t\right)$ , where learned function $f_{\theta }$ captures biological transition rules (Chen et al., 2018b; Grathwohl et al., 2019). ODE-based models excel at trajectory inference by enforcing temporal consistency. Cells at intermediate pseudotimes must lie on smooth paths connecting progenitor and terminal states (Bergen et al., 2020; Qiu et al., 2022). However, these methods typically operate on pre-computed embeddings from PCA or scVI, inheriting any distortions from upstream dimensionality reduction (Setty et al., 2019; Lange et al., 2022). scTour (Li, 2023) integrates VAE training with ODE constraints but lacks geometric regularization to preserve hierarchical structure. We hypothesized that combining hyperbolic geometric principles with neural ODE dynamics within a unified generative framework could yield complementary regularization.

### 1.3 LAIOR: lorentz attentive interpretable ODE-regularized VAE

**FIGURE 1:**
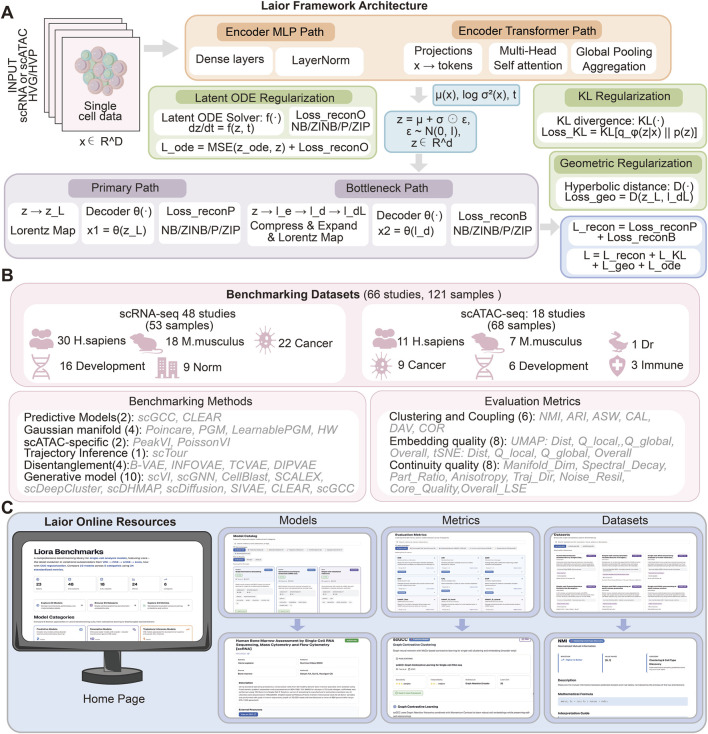
LAIOR architecture, benchmarking framework, and online resources. **(A)** Model architecture showing Transformer encoder with self-attention, dual-path latent representation (primary + bottleneck), latent ODE regularization, hyperbolic geometry constraints, and parallel decoder reconstruction. **(B)** Benchmarking framework: 118 datasets (53 scRNA-seq +65 scATAC-seq), 23 baseline methods across six paradigms, 22 evaluation metrics covering clustering, embedding quality, and continuity. **(C)** Interactive web portal with four sections: Home, Models, Metrics, and Datasets.

We present LAIOR (Lorentz attentive interpretable ODE-regularized VAE), a deep generative framework that addresses the local–global trade-off through three architectural innovations (Figure 1A). First, *Lorentz geometric regularization* aligns latent representations with hyperbolic structure *without* embedding the entire latent space in hyperbolic manifolds. LAIOR maintains a Euclidean latent space for computational stability while using Lorentzian distance penalties to preserve hierarchical relationships during training, thereby avoiding numerical instabilities while capturing the exponential capacity of negative curvature geometries (Nickel and Kiela, 2017).

Second, a dual-path information bottleneck architecture comprises a primary high-dimensional pathway and a compressed bottleneck pathway. The bottleneck does not enforce statistical independence as in β -VAE variants (Higgins et al., 2017), but instead, it captures *shared biological factors* through reconstruction-consistency regularization. Our architectural approach builds upon interpretable VAE concepts (Fu et al., 2025) while introducing geometric and dynamical regularization. By penalizing geometric distance between primary and bottleneck representations in Lorentz space, the architecture preserves coordinated gene expression patterns that define biological processes such as cell-cycle phase, lineage identity, and stress responses (Locatello et al., 2019).

Third, *neural ODE regularization* is integrated directly into VAE training. The encoder predicts pseudotime for each cell, and an ODE solver computes trajectory-consistent latent states by integrating learned dynamics $f_{\theta }\left(z,t\right)$ . A reconstruction loss penalizes deviations between encoder outputs and ODE predictions, enforcing temporal smoothness. In ablation experiments (Section 3.2), Lorentz regularization alone improved global topology but left trajectory inference unstable under biological noise. ODE constraints stabilize manifold estimation, improving noise resilience, trajectory coherence, and overall latent quality over geometric regularization alone.

We benchmarked LAIOR on 53 scRNA-seq and 65 scATAC-seq datasets spanning human and mouse tissues, cancer, immune responses, and development (Figure 1B; full dataset descriptions are available at https://PeterPonyu.github.io/liora-ui and in the Supplementary Material). We compared LAIOR with 23 baseline methods across six paradigms: classical methods (PCA, ICA, factor analysis), generative models (scVI, scGNN, scDiffusion, siVAE), contrastive learning (scGCC, CLEAR), hyperbolic embeddings (Poincaré maps, PGM, learnable Poincaré Gaussian mixture (LPGM), hyperbolic wrapped normal (HWN), scDHMAP), factor-based VAEs ( β -VAE, InfoVAE, TC-VAE, DIP-VAE), and modality-specific methods (PeakVI, PoissonVI, scTour). Because our focus is on representation learning, batch correction tools such as Harmony (Korsunsky et al., 2019) and Seurat integration (Stuart et al., 2019) were not included.

We evaluated performance with 22 metrics in three groups: (i) clustering quality (normalized mutual information, adjusted Rand index, silhouette coefficient, Calinski–Harabasz index, Davies–Bouldin index, and correlation preservation), (ii) embedding fidelity for UMAP and t-SNE projections (distance correlation, local/global/overall quality), and (iii) manifold continuity (intrinsic dimensionality, spectral decay, participation ratio, anisotropy, trajectory directionality, noise resilience, core quality, and overall latent quality). As in standard single-cell workflows, the clustering analyses are anchored by labels derived from unsupervised analysis followed by marker-based curation (Kiselev et al., 2019; Abdelaal et al., 2019).

Ablation studies show that the full model performs best when geometric, bottleneck, and dynamical regularization are used together. Across the benchmark, LAIOR improves clustering-related structure, embedding fidelity, and continuity while remaining interpretable at the latent factor level. Analyses of human bone marrow and mouse pancreatic endocrinogenesis link the learned factors to cell cycle, lineage commitment, and functional programs, and trajectory analyses on hematopoietic differentiation and perturbation datasets show that LAIOR can resolve branching structure while separating biological dynamics from technical confounding.

LAIOR is designed to work across scRNA-seq and scATAC-seq without architectural changes, although it is not intended for paired multi-omics integration. An interactive web portal (Figure 1C) provides access to the models, datasets, metrics, and trained weights to support reproducibility and reuse.

## 2 Materials and methods

### 2.1 Model architecture overview

LAIOR (Lorentz attentive interpretable ODE-regularized VAE) is a deep generative model integrating variational inference, geometric regularization, information bottleneck theory, and continuous dynamics modeling for single-cell trajectory analysis. The architecture comprises four core components: (1) a Transformer-based encoder with self-attention mechanisms for capturing long-range gene dependencies, (2) Lorentz manifold geometric regularization, (3) an information bottleneck for coordinated biological program discovery, and (4) neural ODE-based trajectory regularization.

#### 2.1.1 Module dataflow

**FIGURE 2:**
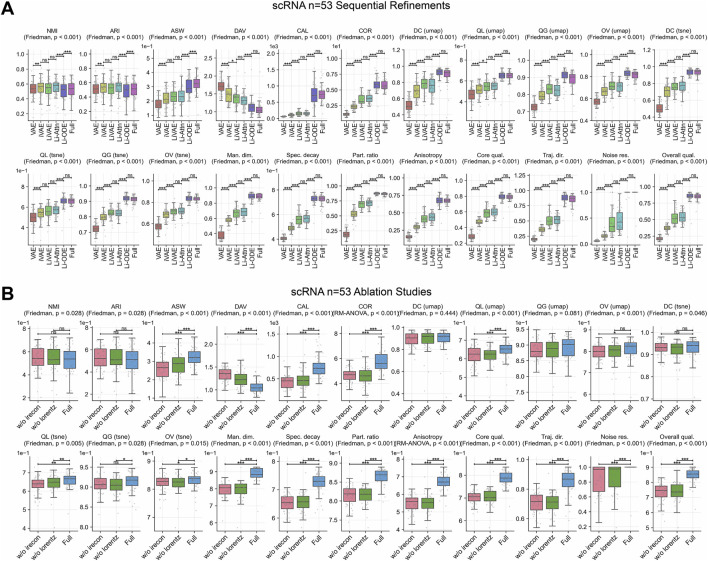
Module and regularization combination analysis: sequential refinement and targeted ablation across 53 scRNA-seq datasets. **(A)** Progressive component additions: VAE (baseline) → iVAE (information bottleneck) → LiVAE (Lorentz geometry) → Li-Attn (Transformer encoder) → Li-ODE (MLP encoder with ODE regularization) → LAIOR (Transformer encoder with full integration). Note: Li-Attn and Li-ODE represent parallel architectural branches exploring encoder architectures; LAIOR combines Transformer attention with a complete regularization framework. Evaluated across 22 metrics defined in Materials and Methods. Friedman tests confirm significant differences across configurations (p<0.001) . **(B)** Ablation study comparing w/o Lorentz (LAIOR minus geometric regularization), w/o irecon (LAIOR minus information bottleneck), and full LAIOR. Statistical comparisons *via* Wilcoxon signed-rank tests with Bonferroni correction: * (p<0.05) , ** (p<0.01) , *** (p<0.001) , ns (not significant).

The four components are arranged in a single forward pass with parallel regularization branches rather than sequential stages. Explicitly, for each input cell $x\in R^{d}$ : (i) the *encoder* produces a pair of Gaussian parameters (μ(x),σ(x)) from which the primary latent $z∼N\left(\mu ,\sigma^{2}I\right)\in R^{l}$ is sampled; (ii) the same encoder output feeds a *bottleneck projection* producing the compressed latent $l_{d}\in R^{i}$ (i≪l) ; (iii) z and $l_{d}$ are mapped to the Lorentz hyperboloid *via* the exponential map, and the *Lorentz loss* $L_{\text{geom}}=d_{H}\left(z_{L},l_{dL}\right)$ regularizes their geometric alignment; (iv) the *ODE branch* integrates the latent dynamics $dz/dt=f_{\theta }\left(z,t\right)$ over the encoder-predicted pseudotime, and the *ODE loss* $L_{\text{ODE}}$ penalizes deviations between encoder-produced latents and ODE-integrated latents; (v) finally, the *decoder* consumes both z and $l_{d}$ through parallel reconstruction heads producing per-cell gene-wise likelihood parameters. The Lorentz, ODE, KL, and information bottleneck terms are combined *via* Equation 43 as independent regularizers on a single shared latent, not as sequential downstream modules. This clarifies that removing any one regularization term (ablation) simply deletes one additive loss component without changing the encoder/decoder topology, exactly as reported in Figure 2.

#### 2.1.2 Symbol glossary

For reference, Table 1 consolidates the core symbols used throughout the Methods section.

**TABLE 1:** Symbol glossary for LAIOR.

Symbol	Space/Type	Meaning
x	$R^{d}$ (counts)	Raw gene expression vector for 1 cell (input to encoder)
μ,σ	$R^{l}$ each	Posterior mean and standard deviation output by the encoder
z	$R^{l}$	Primary latent sample drawn from $N\left(\mu ,\sigma^{2}I\right)$
$l_{d}$	$R^{i}$ , i<l	Compressed information bottleneck latent, derived from the same encoder
$z_{L},l_{dL}$	$H^{l}$	Hyperbolic projections of z and $l_{d}$ (exponential map)
$v_{\text{tangent}}$	$R^{l+1}$	Clipped tangent-space vector fed into the exponential map
t	[0,1]	Encoder-predicted self-supervised pseudotime scalar per cell
$f_{\theta }$	$R^{l}\times R\to R^{l}$	ODE drift function (neural network with parameters θ )
$z_{\text{ODE}}\left(t\right)$	$R^{l}$	Latent state obtained by integrating $f_{\theta }$ from initial condition
$f_{\phi }$	Encoder network	Encoder parameters (bundled under ϕ )
$g_{\psi }$	Decoder network	Decoder parameters (bundled under ψ )
$\lambda_{\text{recon/irecon/geom/ODE}},\;\beta$	$R_{\geq 0}$	Loss-term weighting hyperparameters (Equation 43)
k,e	Integers	Transformer token count (k) and embedding dim. (e)
n	Integer	Number of Transformer encoder layers

#### 2.1.3 Encoder architecture

The encoder maps high-dimensional gene expression data $x\in R^{d}$ to a latent probability distribution $q_{\phi }\left(z|x\right)$ , where d denotes the number of genes or chromatin features. LAIOR implements a Transformer-based architecture as the primary encoder, with a multilayer perceptron (MLP) fallback option for computational efficiency on smaller datasets.

##### 2.1.3.1 Transformer encoder (default)

For attention-based encoding, input features are projected to a sequence of k tokens (Equation 1):

$$X_{\text{seq}}=\text{Reshape}\left(W_{\text{proj}}x+b_{\text{proj}},\left(k,e\right)\right),$$ (1)

where $W_{\text{proj}}\in R^{ke\times d}$ , $b_{\text{proj}}\in R^{ke}$ , and e is the embedding dimension, yielding $X_{\text{seq}}\in R^{k\times e}$ . This sequence is processed through n Transformer layers, where each layer j∈{1,…,n} (Equations 2, 3).

$$X_{\text{attn}}^{\left(j\right)}=\text{LayerNorm}\left(X_{\text{seq}}^{\left(j-1\right)}+\text{MultiHeadAttention}\left(X_{\text{seq}}^{\left(j-1\right)}\right)\right),$$ (2)

$$X_{\text{seq}}^{\left(j\right)}=\text{LayerNorm}\left(X_{\text{attn}}^{\left(j\right)}+\text{FFN}\left(X_{\text{attn}}^{\left(j\right)}\right)\right),$$ (3)

where $X_{\text{seq}}^{\left(0\right)}=X_{\text{seq}}$ . Multi-head self-attention with m heads computes Equation 4:

$$\text{MultiHeadAttention}\left(X\right)=\text{Concat}\left({\text{head}}_{1},\ldots ,{\text{head}}_{m}\right)W^{O},$$ (4)

where each head is defined using Equation 5:

$${\text{head}}_{i}=\text{softmax}\left(\frac{Q_{i}K_{i}^{⊤}}{\sqrt{e/m}}\right)V_{i},$$ (5)

with $Q_{i}=XW_{i}^{Q}$ , $K_{i}=XW_{i}^{K}$ , $V_{i}=XW_{i}^{V}$ , and $W_{i}^{Q},W_{i}^{K},W_{i}^{V}\in R^{e\times \left(e/m\right)}$ . The position-wise feedforward network applies (Equation 6):

$$\text{FFN}\left(X\right)=\text{ReLU}\left(XW_{1}^{\text{FFN}}+b_{1}^{\text{FFN}}\right)W_{2}^{\text{FFN}}+b_{2}^{\text{FFN}}.$$ (6)

The final output $X_{\text{seq}}^{\left(n\right)}\in R^{k\times e}$ is aggregated *via* mean pooling and projected to latent parameters using Equation 7:

$$\left[\mu ,\log\sigma^{2}\right]=W_{\text{out}}\cdot \frac{1}{k}\overset{k}{\underset{i=1}{\sum }}X_{\text{seq},i}^{\left(n\right)}+b_{\text{out}},$$ (7)

where $W_{\text{out}}\in R^{2l\times e}$ and $b_{\text{out}}\in R^{2l}$ .

##### 2.1.3.2 MLP encoder (alternative)

The default architecture consists of two hidden layers with ReLU activations and layer normalization. Let h denote the hidden dimension and let l denote the latent dimension. The encoder is defined by Equations 8–10:

$$h_{1}=\text{ReLU}\left(\text{LayerNorm}\left(W_{1}x+b_{1}\right)\right),$$ (8)

$$h_{2}=\text{ReLU}\left(\text{LayerNorm}\left(W_{2}h_{1}+b_{2}\right)\right),$$ (9)

$$\left[\mu ,\log\sigma^{2}\right]=W_{3}h_{2}+b_{3},$$ (10)

where $W_{1}\in R^{h\times d}$ , $W_{2}\in R^{h\times h}$ , $W_{3}\in R^{2l\times h}$ , and $b_{1},b_{2}\in R^{h}$ , $b_{3}\in R^{2l}$ are bias vectors. The output is split into mean $\mu \in R^{l}$ and log-variance $\log\sigma^{2}\in R^{l}$ .

##### 2.1.3.3 Architecture selection

All benchmarking experiments reported in Results used the Transformer encoder unless explicitly stated otherwise. The Li-ODE architectural variant (Section 3.2) employed the MLP encoder to isolate ODE regularization contributions independent of attention mechanisms. The complete LAIOR model integrates Transformer encoding with the full regularization framework.

Both encoders apply numerical stabilization to outputs (Equations 11, 12).

$$\mu \leftarrow \text{clamp}\left(\mu ,-10,10\right),$$ (11)

$$\sigma =\text{clamp}\left(\text{softplus}\left(\log\sigma^{2}\right)+10^{-6},10^{-6},5.0\right),$$ (12)

where $\text{softplus}\left(x\right)=\log\left(1+e^{x}\right)$ . The posterior distribution $q_{\phi }\left(z|x\right)=N\left(\mu ,\text{diag}\left(\sigma^{2}\right)\right)$ is sampled *via* the reparameterization trick defined in Equation 13:

$$z=\mu +\sigma ⊙\epsilon ,\;\epsilon ∼N\left(0,I_{l}\right),$$ (13)

where ⊙ denotes element-wise multiplication.

For temporal modeling, the encoder includes a pseudotime prediction head defined by Equation 14:

$$t=\sigma_{\text{sig}}\left(w_{t}^{⊤}h_{2}+b_{t}\right)\in \left[0,1\right],$$ (14)

where $w_{t}\in R^{h}$ , $b_{t}\in R$ , and $\sigma_{\text{sig}}\left(\cdot \right)$ is the sigmoid activation function.

#### 2.1.4 Lorentz geometric regularization

Latent codes are embedded onto the Lorentz model of hyperbolic space, defined as Equation 15. This manifold-aware optimization context follows Riemannian adaptive optimization methods (Bécigneul and Ganea, 2019).

$$H^{n}=\left\{x\in R^{n+1}:{\left\langle x,x\right\rangle }_{L}=-1,x_{0}>0\right\},$$ (15)

where the Lorentzian inner product is defined by Equation 16:

$${\left\langle x,y\right\rangle }_{L}=-x_{0}y_{0}+\overset{n}{\underset{i=1}{\sum }}x_{i}y_{i}.$$ (16)

##### 2.1.4.1 Exponential map

Latent codes $z\in R^{l}$ are mapped from the tangent space at the origin $o={\left[1,0,\ldots ,0\right]}^{⊤}\in H^{l}$ to the manifold. The tangent space is defined by Equation 17:

$$T_{o}H^{l}=\left\{v\in R^{l+1}:v_{0}=0\right\}.$$ (17)

Given latent code $z\in R^{l}$ , we construct the tangent vector (Equations 18, 19).

$$z_{\text{clip}}=\text{clamp}\left(z,-5,5\right),$$ (18)

$$v_{\text{tangent}}={\left[0,z_{\text{clip},1},z_{\text{clip},2},\ldots ,z_{\text{clip},l}\right]}^{⊤}\in R^{l+1}.$$ (19)

Compute the spatial norm using Equation 20:

$$r=\text{clamp}\left(\sqrt{\overset{l}{\underset{i=1}{\sum }}v_{\text{tangent},i}^{2}},0,15\right).$$ (20)

The exponential map $\exp_{o}:T_{o}H^{l}\to H^{l}$ is defined by Equation 21:

$$\exp_{o}\left(v_{\text{tangent}}\right)=\left[\begin{array}{c} \cosh\left(r\right)\\ \sinh\left(r\right)\cdot \frac{v_{\text{spatial}}}{r} \end{array}\right],$$ (21)

where $v_{\text{spatial}}={\left[v_{\text{tangent},1},\ldots ,v_{\text{tangent},l}\right]}^{⊤}\in R^{l}$ . For $r<10^{-8}$ , we set $v_{\text{spatial}}=0$ to avoid division by zero.

##### 2.1.4.2 Hyperbolic distance

The distance between points $x,y\in H^{l}$ is defined by Equation 22:

$$d_{H}\left(x,y\right)=\text{arccosh}\left(\max\left(1+10^{-8},-{\left\langle x,y\right\rangle }_{L}\right)\right).$$ (22)

For numerical stability when $-{\left\langle x,y\right\rangle }_{L}>10^{4}$ , we use the asymptotic approximation defined by Equation 23:

$$d_{H}\left(x,y\right)\approx \log\left(2\cdot \left(-{\left\langle x,y\right\rangle }_{L}\right)\right).$$ (23)

Manifold embeddings for the primary and bottleneck latents are defined by Equations 24, 25:

$$z_{L}=\exp_{o}\left({\left[0,\text{clamp}\left(z,-5,5\right)\right]}^{⊤}\right)\in H^{l},$$ (24)

$$l_{dL}=\exp_{o}\left({\left[0,\text{clamp}\left(l_{d},-5,5\right)\right]}^{⊤}\right)\in H^{l}.$$ (25)

#### 2.1.5 Information bottleneck

The information bottleneck pathway compresses the latent code through a lower-dimensional bottleneck with dimension i<l , designed to extract shared biological programs. Unlike disentanglement approaches that enforce statistical independence, LAIOR’s bottleneck preserves coordinated variation patterns through geometric consistency regularization, enabling the capture of interconnected biological processes such as cell-cycle coordination, lineage-coupled differentiation programs, and stress-response modules.

##### 2.1.5.1 Compression

Linear encoding to bottleneck (Equation 26):

$$l_{e}=W_{e}z+b_{e},$$ (26)

where $W_{e}\in R^{i\times l}$ , $b_{e}\in R^{i}$ , and $l_{e}\in R^{i}$ .

##### 2.1.5.2 Decompression

Linear decoding from bottleneck (Equation 27):

$$l_{d}=W_{d}l_{e}+b_{d},$$ (27)

where $W_{d}\in R^{l\times i}$ , $b_{d}\in R^{l}$ , and $l_{d}\in R^{l}$ .

##### 2.1.5.3 Geometric integration

The decompressed latent is mapped to the Lorentz manifold using Equation 28:

$$l_{dL}=\exp_{o}\left({\left[0,\text{clamp}\left(l_{d},-5,5\right)\right]}^{⊤}\right)\in H^{l}.$$ (28)

Both primary latent z and bottleneck latent $l_{d}$ are independently decoded through the same decoder network to produce parallel reconstructions.

#### 2.1.6 Neural ODE latent regularization

For temporal modeling, latent dynamics are governed by the following ordinary differential equation (Equation 29):

$$\frac{dz}{dt}=f_{\theta }\left(z,t\right),$$ (29)

where $z\left(t\right)\in R^{l}$ represents the latent state at pseudotime t∈[0,1] , and $f_{\theta }:R^{l}\times R\to R^{l}$ is a parametric function.

##### 2.1.6.1 ODE function

The time-conditioned ODE function is a two-layer neural network with hidden dimension $h_{\text{ode}}$ (Equations 30, 31).

$$h_{\text{ode}}=\text{ELU}\left(W_{1}\left[z;t\right]+b_{1}\right),$$ (30)

$$f_{\theta }\left(z,t\right)=W_{2}h_{\text{ode}}+b_{2},$$ (31)

where $\left[z;t\right]\in R^{l+1}$ denotes concatenation, $W_{1}\in R^{h_{\text{ode}}\times \left(l+1\right)}$ , $b_{1}\in R^{h_{\text{ode}}}$ , $W_{2}\in R^{l\times h_{\text{ode}}}$ , $b_{2}\in R^{l}$ , and ELU(x)=x if x>0 and $\text{ELU}\left(x\right)=\alpha \left(e^{x}-1\right)$ if x≤0 , with α=1.0 .

##### 2.1.6.2 Trajectory integration

For N cells with pseudotime predictions $\left\{t_{1},\ldots ,t_{N}\right\}\subset \left[0,1\right]$ , we sort cells by pseudotime and remove duplicates to obtain a strictly increasing sequence ${\tilde{t}}_{1}<{\tilde{t}}_{2}<\cdots <{\tilde{t}}_{M}$ where M≤N .

Setting the initial condition $z_{0}=z\left({\tilde{t}}_{1}\right)$ , we solve the initial value problem using Equation 32:

$$\left\{\begin{array}{c} \frac{dz}{dt}=f_{\theta }\left(z,t\right),\\ z\left({\tilde{t}}_{1}\right)=z_{0}, \end{array}\right.$$ (32)

using the fourth-order Runge–Kutta (RK4) method. At step n , we compute (Equations 33–37)

$$k_{1}=f_{\theta }\left(z_{n},t_{n}\right),$$ (33)

$$k_{2}=f_{\theta }\left(z_{n}+\frac{\Delta t}{2}k_{1},t_{n}+\frac{\Delta t}{2}\right),$$ (34)

$$k_{3}=f_{\theta }\left(z_{n}+\frac{\Delta t}{2}k_{2},t_{n}+\frac{\Delta t}{2}\right),$$ (35)

$$k_{4}=f_{\theta }\left(z_{n}+\Delta tk_{3},t_{n}+\Delta t\right),$$ (36)

$$z_{n+1}=z_{n}+\frac{\Delta t}{6}\left(k_{1}+2k_{2}+2k_{3}+k_{4}\right),$$ (37)

where $\Delta t={\tilde{t}}_{n+1}-{\tilde{t}}_{n}$ is the step size, to obtain ODE-solved latents $\left\{z_{\text{ODE}}\left({\tilde{t}}_{1}\right),z_{\text{ODE}}\left({\tilde{t}}_{2}\right),\ldots ,z_{\text{ODE}}\left({\tilde{t}}_{M}\right)\right\}$ .

This creates three parallel reconstruction pathways.

1. Primary path: Reconstruction using encoder latent z

2. Bottleneck path: Reconstruction using decompressed latent $l_{d}$

3. ODE path: Reconstruction using trajectory-regularized latent $z_{\text{ODE}}$

#### 2.1.7 Decoder architecture

The decoder reconstructs gene expression from latent codes *via* a two-layer feedforward network given input latent code $z_{\text{in}}\in R^{l}$ (which may be z , $l_{d}$ , or $z_{\text{ODE}}$ ) (Equations 38–40).

$$g_{1}=\text{ReLU}\left(\text{LayerNorm}\left(W_{1}^{\left(d\right)}z_{\text{in}}+b_{1}^{\left(d\right)}\right)\right),$$ (38)

$$g_{2}=\text{ReLU}\left(\text{LayerNorm}\left(W_{2}^{\left(d\right)}g_{1}+b_{2}^{\left(d\right)}\right)\right),$$ (39)

$$\pi =\text{softmax}\left(W_{3}^{\left(d\right)}g_{2}+b_{3}^{\left(d\right)}\right),$$ (40)

where $W_{1}^{\left(d\right)}\in R^{h\times l}$ , $W_{2}^{\left(d\right)}\in R^{h\times h}$ , $W_{3}^{\left(d\right)}\in R^{d\times h}$ , and $b_{1}^{\left(d\right)},b_{2}^{\left(d\right)}\in R^{h}$ , $b_{3}^{\left(d\right)}\in R^{d}$ . The softmax output $\pi \in \Delta^{d-1}$ (the (d−1) -dimensional probability simplex) serves as the mean parameter for likelihood functions.

A learned per-gene dispersion parameter $\theta \in R^{d}$ models overdispersion for negative binomial likelihoods. For zero-inflated models, an auxiliary network predicts zero-inflation logits (Equations 41, 42).

$$h_{\text{drop}}=\text{ReLU}\left(W_{\text{drop},1}z_{\text{in}}+b_{\text{drop},1}\right),$$ (41)

$$\rho =W_{\text{drop},2}h_{\text{drop}}+b_{\text{drop},2},$$ (42)

where $W_{\text{drop},1}\in R^{h\times l}$ , $b_{\text{drop},1}\in R^{h}$ , $W_{\text{drop},2}\in R^{d\times h}$ , $b_{\text{drop},2}\in R^{d}$ , and $\rho \in R^{d}$ give logit-space dropout probabilities.

Weight matrices are initialized using Xavier normal initialization. Bias terms are initialized to 0.01.

### 2.2 Training procedure and optimization

#### 2.2.1 Data preprocessing

Raw count matrices were extracted and validated for integer-valued counts. Log-transformation $\log_{2}\left(x+1\right)$ was applied, followed by adaptive clipping. High-sparsity datasets (>95% zeros) were clipped to [−5,5] ; all other datasets were clipped to [−10,10] . The two clipping ranges reflect the empirical distributions of post- $\log_{2}$ expression values: on typical single-cell data, more than 99.5% of non-zero $\log_{2}\left(x+1\right)$ values fall within [−10,10] , and on high-sparsity profiles where the signal is concentrated in fewer genes, the tighter [−5,5] window better suppresses outlier-driven gradient variance without truncating biologically meaningful variation. This clipping step is distinct from the z -score outlier clipping at ±10 standard deviations applied internally by the training data loader (Section 2.5.2): the former acts on the raw $\log_{2}$ -scale values before normalization, the latter acts on standardized values during batch construction. Data were partitioned into training (70%), validation (15%), and test (15%) sets at the *cell* level with stratified random sampling (random seed 42), ensuring that each cell type is proportionally represented across splits; a complementary dataset-level held-out-dataset protocol, in which entire datasets are excluded from training and evaluated in zero-shot mode, is reported in Supplementary Material.

#### 2.2.2 Optimization

Model parameters were optimized using the Adam optimizer with learning rate $\eta =10^{-4}$ , batch size B=128 , gradient clipping at maximum norm 1.0, and early stopping with patience of 25 epochs based on validation loss. For benchmarking experiments, datasets were subsampled to 3,000 cells.

#### 2.2.3 Loss function

The total loss function is (Equation 43):

$$L_{\text{total}}=\lambda_{\text{recon}}L_{\text{recon}}+\lambda_{\text{irecon}}L_{\text{irecon}}+\lambda_{\text{geom}}L_{\text{geom}}+\beta L_{\text{KL}}+\lambda_{\text{ODE}}L_{\text{ODE}},$$ (43)

where $\lambda_{\text{recon}},\lambda_{\text{irecon}},\lambda_{\text{geom}},\beta ,\lambda_{\text{ODE}}$ are weighting hyperparameters.

##### 2.2.3.1 Package defaults vs. per-experiment values

For clarity, we note that the published LAIOR Python package constructor exposes these loss weights as keyword arguments with *intentionally zero defaults* for all regularization terms except KL (β=1.0) and the primary reconstruction $\left(\lambda_{\text{recon}}=1.0\right)$ . Specifically, the package defaults are $\lambda_{\text{irecon}}=\lambda_{\text{geom}}=\lambda_{\text{dip}}=\lambda_{\text{tc}}=\lambda_{\text{info}}=0$ , reflecting a deliberate “bottom-up ablation” design in which the user must be explicitly enable each regularizer. This design supports the sequential refinement analysis reported in Figure 2: starting from a plain VAE (all extras set to 0) and adding terms one at a time, it isolates each component’s contribution. The *actual values used in each experiment reported in this article* are not the package defaults: full LAIOR uses $\lambda_{\text{irecon}}=1.0$ and $\lambda_{\text{geom}}=5.0$ (i.e., the “high curvature” setting identified in Section 3.4) unless otherwise noted. Hyperparameter sweeps in Section 3.4 explicitly vary these values. There is therefore no discrepancy between the source code and the article: the zero entries in the package signature are the starting points of the ablation series, not the final values.

##### 2.2.3.2 Reconstruction loss

Primary reconstruction loss is defined by Equation 44:

$$L_{\text{recon}}=-E_{q_{\phi }\left(z|x\right)}\left[\log p_{\theta }\left(x|z\right)\right].$$ (44)

Information bottleneck reconstruction loss is defined by Equation 45:

$$L_{\text{irecon}}=-E_{q_{\phi }\left(z|x\right)}\left[\log p_{\theta }\left(x|l_{d}\right)\right].$$ (45)

Predicted rates are scaled by library size: $\mu_{j}=\pi_{j}\cdot s$ where $s=\sum_{j=1}^{d}x_{j}$ is the total count for the cell.

For the negative binomial (NB) likelihood, the log-probability is defined by Equation 46:

$$\begin{array}{c} \log p\left(x|\mu ,\theta \right)=\overset{d}{\underset{j=1}{\sum }}[\log\Gamma \left(x_{j}+\theta_{j}\right)-\log\Gamma \left(\theta_{j}\right)-\log\Gamma \left(x_{j}+1\right)+\theta_{j}\log\frac{\theta_{j}}{\theta_{j}+\mu_{j}}+x_{j}\log\frac{\mu_{j}}{\theta_{j}+\mu_{j}}], \end{array}$$ (46)

where $\theta_{j}=\exp\left({\text{disp}}_{j}\right)$ with learned dispersion parameters ${\text{disp}}_{j}$ .

Zero-inflated negative binomial (ZINB) extends NB *via* (Equations 47, 48)

$$p\left(x_{j}=0\right)=\pi_{\text{drop},j}+\left(1-\pi_{\text{drop},j}\right)\cdot \text{NB}\left(0|\mu_{j},\theta_{j}\right),$$ (47)

$$p\left(x_{j}>0\right)=\left(1-\pi_{\text{drop},j}\right)\cdot \text{NB}\left(x_{j}|\mu_{j},\theta_{j}\right),$$ (48)

where $\pi_{\text{drop},j}=\sigma_{\text{sig}}\left(\rho_{j}\right)$ . Poisson and zero-inflated Poisson (ZIP) likelihoods follow analogously with $\theta_{j}\to \infty$ .

##### 2.2.3.3 Geometric regularization

The distance penalty on the Lorentz manifold is defined by Equation 49:

$$L_{\text{geom}}=d_{H}\left(z_{L},l_{dL}\right).$$ (49)

##### 2.2.3.4 KL divergence

Between posterior $q_{\phi }\left(z|x\right)=N\left(\mu ,\text{diag}\left(\sigma^{2}\right)\right)$ and standard Gaussian prior $p\left(z\right)=N\left(0,I_{l}\right)$ (Equation 50):

$$L_{\text{KL}}=\frac{1}{2}\overset{l}{\underset{j=1}{\sum }}\left(\mu_{j}^{2}+\sigma_{j}^{2}-\log\sigma_{j}^{2}-1\right).$$ (50)

##### 2.2.3.5 ODE trajectory consistency

The mean squared error between encoder latents and ODE-predicted latents is defined by Equation 51:

$$L_{\text{ODE}}=\frac{1}{M}\overset{M}{\underset{i=1}{\sum }}\|z\left({\tilde{t}}_{i}\right)-z_{\text{ODE}}\left({\tilde{t}}_{i}\right){\|}_{2}^{2}.$$ (51)

The default weight coefficients are $\lambda_{\text{recon}}=1.0$ , $\lambda_{\text{irecon}}=1.0$ , $\lambda_{\text{geom}}=5.0$ , β=1.0 , and $\lambda_{\text{ODE}}=1.0$ unless otherwise specified.

#### 2.2.4 Architecture specifications

##### 2.2.4.1 Default configuration

Transformer encoder: k=32 tokens, embedding dimension e=64 , m=4 attention heads, n=2 layers, FFN hidden dimension 4e . Decoder: two hidden layers (dimension h=128 ) with ReLU activations, input dimension d (dataset-dependent), latent dimension l=10 . Information bottleneck dimension: i=2 . ODE function hidden dimension: $h_{\text{ode}}=64$ .

##### 2.2.4.2 MLP configuration (when applicable)

Encoder/Decoder: two hidden layers (dimension h=128 ); all other hyperparameters are identical to the default.

Numerical stabilization: encoder mean outputs are clamped to [−10,10] , posterior standard deviations are constrained to $\left[10^{-6},5.0\right]$ , and Lorentz tangent-space inputs are clamped to [−5,5] before the exponential map.

#### 2.2.5 Pseudotime prediction and ODE integration

For ODE-augmented models, the encoder predicts pseudotime *via* a sigmoid-activated linear head, normalized to [0,1] . We emphasize that pseudotime prediction is *self-supervised*: no externally computed pseudotime label (e.g., diffusion pseudotime, Monocle) is provided as a target. Instead, pseudotime values emerge from the joint optimization of the reconstruction, KL, Lorentz, information bottleneck, and neural ODE trajectory-consistency losses (Equation 43). The ODE reconstruction loss $L_{\text{ODE}}=\frac{1}{M}\sum_{i}\|z\left({\tilde{t}}_{i}\right)-z_{\text{ODE}}\left({\tilde{t}}_{i}\right){\|}_{2}^{2}$ penalizes inconsistencies between encoder-produced latents and ODE-integrated latents, implicitly selecting pseudotime orderings that admit a smooth trajectory. This makes LAIOR directly applicable to new datasets without requiring external pseudotime annotation at inference time. Cells are sorted by pseudotime with duplicate time values removed, and the ODE is solved using RK4 integration on the CPU for numerical stability.

#### 2.2.6 Training protocol

Models were trained with validation evaluation every five epochs. Early stopping was triggered when validation loss failed to improve for 25 consecutive validation checks (equivalent to 125 training epochs), at which point the best checkpoint was restored. This patience parameter balances exploration of the loss landscape with computational efficiency.

#### 2.2.7 Computational environment

All experiments were conducted on a single NVIDIA RTX 5090 GPU (24 GB VRAM) using PyTorch 2.0 with single-precision floating-point arithmetic (FP32). Datasets were subsampled to 3,000 cells for benchmarking, with a batch size of 128.

### 2.3 Evaluation metrics

#### 2.3.1 Clustering quality metrics

We assessed biological population structure using five standard metrics and one novel metric.

##### 2.3.1.1 Standard metrics

Normalized mutual information (NMI) and adjusted Rand index (ARI) measure agreement between predicted clusters and reference pseudo-labels, with values near 1 indicating strong correspondence. We adopt a unified unsupervised evaluation protocol because single-cell transcriptomic data are inherently unsupervised. Cell-type annotations depend on the specific clustering algorithm, resolution parameter, and marker-gene panel used by each laboratory, and no universally accepted ground truth exists. Reference pseudo-labels are generated by applying K -means clustering ( K=l , where l is the latent dimensionality) to the preprocessed expression matrix *before* any representation learning method is applied. For each method under evaluation, K -means is independently applied to the method’s latent space, and NMI/ARI quantify how well the method’s latent clusters recover the structure identified in the original expression space. This design ensures that (i) no method-specific annotation bias contaminates the comparison, and (ii) the reference is consistently derived from the same preprocessing pipeline across all datasets. The average silhouette width (ASW) and the Calinski–Harabasz index (CAL) quantify cluster cohesion and separation (higher is better), while the Davies–Bouldin index (DAV) measures average cluster similarity (lower is better). ASW, CAL, and DAV are computed in the method’s latent space using its own K -means assignments, measuring intrinsic latent-space cluster quality independently of the reference. All metrics were computed using standard implementations in scikit-learn.

##### 2.3.1.2 Coupling degree (COR)

We introduce the coupling degree (COR) metric, Equation 52, to quantify preservation of interdependent biological programs:

$$\text{COR}=\frac{1}{l\left(l-1\right)}\overset{l}{\underset{i=1}{\sum }}\overset{l}{\underset{j\neq i}{\sum }}|\rho_{ij}|,$$ (52)

where $\rho_{ij}$ is the Pearson correlation between latent dimensions i and j , and l is the latent space dimensionality. Higher COR values indicate stronger coupling, reflecting coordinated gene expression programs essential for continuous differentiation trajectories.

##### 2.3.1.3 A note on circular reasoning in embedding-quality evaluation

We acknowledge that embedding quality metrics that rely on neighborhood preservation ( $Q_{\text{local}}$ , $Q_{\text{global}}$ , distance correlation) can favor methods whose training objectives already optimize for neighborhood structure, introducing a potential circularity. We mitigate this in three ways. First, clustering metrics (NMI, ARI) compare each method’s K -means assignments against unsupervised reference pseudo-labels derived from the raw data, providing an independent signal that is independent of the method’s training objective. Second, our intrinsic manifold metrics (Section 2.3.3) quantify spectral and geometric properties of the latent representation itself without reference to any neighborhood graph, providing a complementary signal orthogonal to any neighborhood-preserving training objective. Third, cross-dataset consistency, which is the fact that LAIOR’s advantage over baselines is preserved across 53 scRNA-seq, 65 scATAC-seq, and eight application datasets spanning diverse tissues and species, provides strong evidence against circularity: a method benefiting from objective-metric circularity would not systematically outperform on independent intrinsic metrics and independent datasets simultaneously. Batch-effect-aware evaluation metrics further motivate this caution (Büttner et al., 2019).

##### 2.3.1.4 On dataset-level generalization versus within-dataset evaluation

A single-cell dataset represents one biological snapshot: a specific tissue, developmental stage, and experimental condition. Within-dataset evaluation (train/test split at the cell level) measures a model’s ability to reconstruct the manifold of *that particular* snapshot. However, it does not test whether the learned inductive biases transfer to fundamentally different biological systems. True generalization requires consistent performance across diverse datasets spanning different tissues (bone marrow, pancreas, lung, muscle, liver), species (human, mouse), and biological processes (development, cancer, perturbation). Our benchmark evaluates LAIOR across 53 scRNA-seq and 65 scATAC-seq datasets covering this diversity; the additional eight application datasets in this revision further strengthen the generalization claim by including both well-characterized developmental systems (cd34 hematopoiesis, endo pancreatic endocrinogenesis) and novel perturbation contexts (GSE278673 hematopoietic knockout, GSE277292 wildtype/knockout comparison). The fact that LAIOR’s architectural advantages, including Lorentz geometry for hierarchical structure and neural ODE for temporal consistency, transfer across these heterogeneous biological contexts demonstrates dataset-level generalization that cannot be inferred from within-dataset cell-level splits alone.

#### 2.3.2 Dimensionality reduction embedding-quality metrics

We evaluated how effectively latent representations $Z\in R^{n\times l}$ (where n is the number of cells) project to interpretable low-dimensional spaces (UMAP and t-SNE) while preserving biological relationships.

Distance correlation $\left(\rho_{\text{dist}}\right)$ quantifies preservation of pairwise distance relationships using Equation 53:

$$\rho_{\text{dist}}=\rho_{\text{Spearman}}\left(\text{vec}\left(D_{Z}\right),\text{vec}\left(D_{E}\right)\right),$$ (53)

where $D_{Z}\in R^{n\times n}$ and $D_{E}\in R^{n\times n}$ are pairwise Euclidean distance matrices in latent and embedding spaces, vec(⋅) vectorizes the upper triangle, and $\rho_{\text{Spearman}}$ is the Spearman rank correlation coefficient. Higher values indicate better preservation of global structure.

Local quality $\left(Q_{\text{local}}\right)$ and global quality $\left(Q_{\text{global}}\right)$ measure preservation at different scales through co-ranking matrix analysis using Equations 54, 55, respectively.

$$Q_{\text{local}}=\frac{1}{K_{\max}}\overset{K_{\max}}{\underset{K=1}{\sum }}Q_{NX}\left(K\right),$$ (54)

$$Q_{\text{global}}=\frac{1}{n-1-K_{\max}}\overset{n-1}{\underset{K=K_{\max}+1}{\sum }}Q_{NX}\left(K\right),$$ (55)

where $Q_{NX}\left(K\right)$ is the normalized co-ranking quality measure at neighborhood size K , and $K_{\max}$ is the optimal local neighborhood boundary determined automatically *via* the co-ranking framework. Higher values indicate better maintenance of local neighborhoods and large-scale topology, respectively.

Overall embedding quality $\left(Q_{\text{embed}}\right)$ combines the three components using Equation 56:

$$Q_{\text{embed}}=\frac{1}{3}\left(\rho_{\text{dist}}+Q_{\text{local}}+Q_{\text{global}}\right).$$ (56)

#### 2.3.3 Intrinsic manifold quality metrics

We characterized geometric properties of the latent manifold $Z\in R^{n\times l}$ through spectral analysis of the covariance matrix $C=\frac{1}{n-1}Z^{T}Z$ , with eigenvalues $\lambda_{1}\geq \lambda_{2}\geq \cdots \geq \lambda_{l}$ .

Manifold dimensionality $\left(M_{\text{dim}}\right)$ measures representation compactness using Equation 57:

$$M_{\text{dim}}=1-\frac{d_{\text{eff}}-1}{l-1},$$ (57)

where $d_{\text{eff}}$ is the number of principal components explaining 95% variance. Higher values indicate more efficient encoding.

Spectral decay rate $\left(S_{\text{decay}}\right)$ quantifies hierarchical structure clarity using Equation 58:

$$S_{\text{decay}}=\frac{1}{1+e^{\left|\beta \right|}}\cdot \frac{\lambda_{1}}{\sum_{i=1}^{l}\lambda_{i}},$$ (58)

where β is the slope from log-linear regression on log-eigenvalues. Higher values indicate steeper spectrum decay, reflecting a clear hierarchical organization.

Participation ratio $\left(P_{\text{ratio}}\right)$ assesses the balance of variance distribution using Equation 59:

$$P_{\text{ratio}}=\frac{1}{l}\cdot \frac{{\left(\sum_{i=1}^{l}\lambda_{i}\right)}^{2}}{\sum_{i=1}^{l}\lambda_{i}^{2}}.$$ (59)

Higher values indicate more uniform utilization of latent dimensions, preventing dimension collapse.

Anisotropy score $\left(A_{\text{score}}\right)$ quantifies the directional bias strength using Equation 60:

$$A_{\text{score}}=\tanh\left(\frac{\log\left(\lambda_{1}\right)-\log\left(\lambda_{l}+\epsilon \right)}{4}\right),$$ (60)

where $\epsilon =10^{-8}$ . Higher values indicate stronger directional structure along the dominant axes, which are essential for trajectory representation.

Trajectory directionality $\left(T_{\text{dir}}\right)$ measures the dominance of the primary variation axis using Equation 61:

$$T_{\text{dir}}=\frac{\lambda_{1}}{\sum_{i=2}^{l}\lambda_{i}+\epsilon }.$$ (61)

Higher values indicate a single dominant trajectory direction, which is characteristic of linear differentiation processes.

Noise resilience $\left(N_{\text{res}}\right)$ approximates the signal-to-noise ratio using Equation 62:

$$N_{\text{res}}=\min\left(\frac{\sum_{i=1}^{2}\lambda_{i}}{\sum_{i=3}^{l}\lambda_{i}+\epsilon }\cdot \frac{1}{10},1\right).$$ (62)

Higher values indicate robust separation between signal and noise subspaces.

##### 2.3.3.1 Composite scores

We defined two summary metrics: core intrinsic quality integrates fundamental geometric properties using Equation 63,

$$Q_{\text{core}}=\frac{1}{4}\left(M_{\text{dim}}+S_{\text{decay}}+P_{\text{ratio}}+A_{\text{score}}\right),$$ (63)

while overall intrinsic quality incorporates task-oriented components with empirically determined weights (α,β,γ)=(0.5,0.3,0.2) using Equation 64:

$$Q_{\text{overall}}=\alpha \cdot Q_{\text{core}}+\beta \cdot T_{\text{dir}}+\gamma \cdot N_{\text{res}}.$$ (64)

### 2.4 Latent factor attribution and interpretability analysis

#### 2.4.1 Association score calculation

To identify gene signatures associated with each latent dimension, we implemented a dual-pathway attribution method combining correlation and gradient-based sensitivity analysis. For a given gene i and latent factor j , the combined association score $S_{i,j}$ was calculated using Equation 65:

$$S_{i,j}=0.6\cdot \rho \left(x_{i},v_{j}\right)+0.4\cdot {\tilde{g}}_{i,j},$$ (65)

where ρ denotes the Pearson correlation coefficient. For the *encoder pathway* (identifying discriminative markers), $v_{j}$ represents the latent mean $\mu_{j}\in R^{n}$ across all n cells, and gradients $g_{i,j}=\partial \mu_{j}/\partial x_{i}$ were computed *via* backpropagation. For the *decoder pathway* (identifying reconstructive programs), correlations were computed between latent codes $z_{j}\in R^{n}$ and unscaled decoder outputs ${\hat{x}}_{i}\in R^{n}$ , with gradients $g_{i,j}=\partial {\hat{x}}_{i}/\partial z_{j}$ . In both pathways, gradients were normalized $\left({\tilde{g}}_{i,j}\right)$ to the range [−1,1] to preserve directionality (positive/negative regulation).

#### 2.4.2 Latent velocity field computation and visualization

The velocity field $\dot{z}=dz/dt$ that underlies all trajectory visualizations in Figures 7–9 is obtained by a *direct forward evaluation* of the learned ODE drift network $f_{\theta }$ on each encoder output, not by numerical differencing of the ODE-integrated trajectory $z_{\text{ODE}}\left(t\right)$ . For each cell, the encoder produces the posterior quintuple $\left(q_{z},q_{m},q_{s},n,t\right)$ , and the drift $f_{\theta }\left(q_{z},t\right)$ is evaluated in a single forward pass, yielding a per-cell velocity tensor of shape $\left(N_{\text{cells}},d_{\text{latent}}\right)$ . Per-cell latent velocities are then projected onto the two-dimensional UMAP embedding through a cosine-similarity transition matrix computed in latent space.

For visual rendering, the per-cell field is smoothed onto a regular two-dimensional grid by a Gaussian kernel. For each grid point g , the K -nearest embedding neighbors $\left\{e_{k}\right\}$ are weighted using Equation 66:

$$w_{k}=\phi \;\left(\frac{\|g-e_{k}{\|}_{2}}{\sigma \cdot h}\right),$$ (66)

where ϕ is the standard normal density, σ is the mean grid spacing, and h=0.5 is the kernel bandwidth. The smoothed grid velocity is then $V_{\text{grid}}\left(g\right)=\left(\sum_{k}w_{k}v_{k}\right)/\max\left(1,\sum_{k}w_{k}\right)$ . The resulting grid is rendered as a two-dimensional stream field (line density 2, line width 2, arrow style → ). Every trajectory arrow in Figures 7–9 is produced by this single, uniform pipeline; no post-hoc velocity inference tool (e.g., scVelo, VeloVI, VeloVAE, or Monocle3) is applied to any model.

#### 2.4.3 Encoder-pathway perturbation and gene attribution

Encoder-side gradients $g_{i,j}^{\text{enc}}=\partial \mu_{j}/\partial x_{i}$ are computed by automatic differentiation through the complete encoder forward pass, rather than by finite differences or input masking. For each latent dimension j , the scalar objective $J_{j}^{\text{enc}}=\sum_{c}q_{m,c,j}$ , which is the batch-summed posterior mean along j , is back-propagated with respect to the input counts x ; the resulting per-cell gradient is averaged across the cell dimension to produce a per-gene signed gradient. Sweeping $j=0,\ldots ,d_{\text{latent}}-1$ yields the full gradient matrix $G^{\text{enc}}\in R^{N_{\text{genes}}\times d_{\text{latent}}}$ .

The encoder correlation matrix $C^{\text{enc}}\in R^{N_{\text{genes}}\times d_{\text{latent}}}$ is the Pearson correlation between each gene’s raw counts and each latent mean, computed as a single vectorized block of the joint covariance of [X,μ] . Gradients are rescaled to [−1,1] by dividing through by the global absolute maximum of $G^{\text{enc}}$ , which is a *matrix-level* normalization that preserves the sign of regulation. The combined association score used for ranking is the convex combination

$$S_{i,j}^{\text{enc}}=0.6\;\rho_{i,j}^{\text{enc}}+0.4\;{\tilde{g}}_{i,j}^{\text{enc}},$$ (67)

with fixed weights 0.6 and 0.4 applied uniformly across all datasets.

#### 2.4.4 Decoder-pathway perturbation and gene attribution

Decoder-side gradients $g_{i,j}^{\text{dec}}=\partial {\hat{x}}_{i}/\partial z_{j}$ target the *unscaled* decoder output, that is, the raw reconstruction mean before library-size scaling or noise sampling. For each gene i , the scalar objective $J_{i}^{\text{dec}}=\sum_{c}{\hat{\mu }}_{x,c,i}$ is back-propagated against the latent code z , and the resulting per-cell latent gradient is averaged across cells.

The decoder correlation matrix uses $\rho_{i,j}^{\text{dec}}=\text{corr}\left(z_{j},{\hat{x}}_{i}\right)$ . The correlation is between latent codes and the model’s *reconstruction* $\hat{x}$ , not the original input x . This is a deliberate design choice that endows the two pathways with complementary semantics: the encoder pathway is a *regulator score* (which genes drive each latent direction), whereas the decoder pathway is an *effector score* (which genes the model uses to reconstruct each latent direction). Matrix-level [−1,1] normalization and the same convex-combination weights yield (Equation 68)

$$S_{i,j}^{\text{dec}}=0.6\;\rho_{i,j}^{\text{dec}}+0.4\;{\tilde{g}}_{i,j}^{\text{dec}}.$$ (68)

Because encoder gradients flow through x and decoder gradients flow through z , the two top-gene rankings are numerically independent, so their Jaccard overlap is a meaningful diagnostic of encoder–decoder agreement rather than an inherent tautology.

Gene selection, uniqueness constraint, and Gene Ontology biological process (GOBP) enrichment: generation policies of Figures 6, 7. The rankings from Equations 67, 68 feed two complementary analysis pipelines, chosen to match the distinct scientific targets of Figures 6, 7.

Figure 6 shows orthogonal per-factor decomposition (architecture interpretation target). On two well-characterized reference systems (human bone marrow, *cd34*; mouse pancreatic endocrinogenesis, *endo*), we apply a uniqueness constraint that implements a *global* argmax over the full gene × latent score matrix: all gene–latent pairs are flattened and sorted by the signed combined score $S_{i,j}$ in descending order, and each gene is assigned to the single highest-ranked latent in that global ranking, with each latent admitting at most k=50 genes. This produces two non-overlapping gene lists per latent factor (one per pathway), each containing up to 50 genes. The top five latent factors displayed in Panels A–B are ranked by Jaccard overlap between the encoder and decoder gene lists in descending order, so the most interpretable factor always occupies the top row. The GOBP enrichment (Panels C–D) then applies GSEApy (Fang et al., 2023) separately to the encoder and decoder gene lists of the top three Jaccard-sorted factors (two columns per row: “L i enc” and “L i dec”), using MSigDB v2024.1 collections: the c5. go.bp (biological process) collection for the human reference and the m5.go.bp collection for the mouse reference. Enrichment significance is assessed by a hypergeometric (Fisher’s exact) test with Benjamini–Hochberg adjustment; the top 15 terms by adjusted p -value are displayed per panel. The dot size encodes the gene-set overlap fraction, and the dot color encodes the enrichment combined score; a dynamic label-area budget automatically truncates long GOBP names so that every row fits within its panel at the current scaled font size.

Figure 7 shows the encoder–decoder intersection (biological validation target). The same combined-score formulas are used on two previously unseen hematopoietic perturbation cohorts, the *Dapp1* knockout (GSE277292) and the increased risk acute lymphocytic leukemia (IRALL) chemotherapy-induced bone marrow failure (GSE278673). However, but the gene-selection policy changes: for each latent factor, the biological payload is defined as the *intersection* of the top- k encoder and the top- k decoder gene lists,

$$I_{j}={top}_{k}\;\left(S_{\cdot ,j}^{\text{enc}}\right)\cap {top}_{k}\;\left(S_{\cdot ,j}^{\text{dec}}\right).$$

The per-latent intersection sets are then concatenated across factors and passed to a single GOBP enrichment call, producing the Enc/Dec Overlap dot-plot shown in the right column of Panels A and C (15 terms at full panel height). The 5×3 latent factor grid (Panels B and D) again selects the top five latents by descending Jaccard overlap, so the two figures share the same ordering convention.

The two policies are complementary by design: the uniqueness constraint (Figure 6) shows that the encoder and decoder pathways decompose cellular variation into *mutually exclusive* biological modules on reference systems. In contrast, the intersection policy (Figure 7) confirms that a *single core regulatory–effector program* dominates each perturbation cohort on biology unseen during benchmarking.

### 2.5 Datasets and preprocessing

#### 2.5.1 Dataset selection

We curated 118 single-cell datasets from public repositories (Gene Expression Omnibus, GEO): 53 scRNA-seq datasets and 65 scATAC-seq datasets. Raw single-cell count matrices underwent quality control and normalization prior to model training. Both modalities require raw integer counts as input, as the model employs count-based likelihood functions (negative binomial or Poisson).

#### 2.5.2 scRNA-seq preprocessing

The top 3,000 highly variable genes (HVGs) were selected by modeling the mean-variance relationship in count data using the Seurat methodology. For model input, normalized data were obtained by applying $\log_{2}\left(x+1\right)$ transformation followed by z -score standardization with outlier clipping at ±10 standard deviations.

#### 2.5.3 scATAC-seq preprocessing

Term frequency-inverse document frequency (TF-IDF) normalization was applied using Equation 69:

$${\text{TF}-\text{IDF}}_{ij}={\text{TF}}_{ij}\times {\text{IDF}}_{j}\times s,$$ (69)

where the term frequency for cell i and peak j is ${\text{TF}}_{ij}=x_{ij}/\sum_{k}x_{ik}$ , the inverse document frequency is ${\text{IDF}}_{j}=\log\left(1+\frac{N}{n_{j}}\right)$ with N being the total number of cells and $n_{j}$ the number of cells where peak j is accessible, and $s=10^{4}$ is a scaling factor. Highly variable peaks (HVPs) were identified using variance-based selection on TF-IDF normalized values, restricted to peaks with accessibility in 1%–95% of cells. The top 10,000 HVPs were selected as input features.

### 2.6 Baseline methods

To evaluate LAIOR’s performance, we compared it against 22 baseline methods representing state-of-the-art approaches in single-cell analysis. The methods are organized by architectural paradigm.

#### 2.6.1 Contrastive predictive models (encoder-only)

- scGCC (Tian S. W. et al., 2023): Graph attention network with momentum contrast (MoCo) contrastive learning for robust cell embeddings through graph-based neighbor aggregation without reconstruction.
- CLEAR (Han et al., 2022): MoCo-based contrastive learning framework with carefully designed augmentations (masking, noise, jittering) for batch-invariant cell representations without reconstruction.

Deep generative models (VAE/autoencoder with reconstruction).

- scVI (Lopez et al., 2018): Probabilistic variational autoencoder with zero-inflated negative binomial likelihood, establishing the standard for deep generative modeling in single-cell transcriptomics.
- scGNN (Wang et al., 2021): Graph convolutional autoencoder modeling cell–cell relationships *via* left-truncated mixture Gaussian distributions for imputation and clustering.
- SCALEX (Xiong et al., 2022): Batch-aware VAE for online integration, projecting heterogeneous single-cell datasets into batch-invariant common embedding spaces.
- CellBlast (Cao et al., 2020): VAE-based generative model enabling large-scale scRNA-seq database querying *via* unbiased cell embeddings and probabilistic similarity metrics.
- scDeepCluster (Tian et al., 2019): Autoencoder with zero-inflated negative binomial reconstruction and deep embedded clustering (DEC) for joint representation learning and cluster assignment.
- scDHMap (Tian T. et al., 2023): Hyperbolic VAE on Lorentz hyperboloid with ZINB reconstruction and t-SNE repulsion for hierarchical cell-type visualization.
- scDiffusion (Luo et al., 2024): Conditional denoising diffusion probabilistic model for high-quality synthetic single-cell data generation through iterative denoising reconstruction.
- siVAE (Choi et al., 2023): Scalable interpretable VAE with structured latent space, jointly learning cell-wise and feature-wise embeddings to discover interpretable gene modules.

#### 2.6.2 Geometric manifold models (VAE with geometric constraints)

- Poincaré maps (Klimovskaia et al., 2020): Embedding method leveraging the Poincaré disk model of hyperbolic geometry to capture hierarchical structures naturally in single-cell data.
- Hyperbolic wrapped normal (HWN) (Nagano et al., 2019): Probabilistic distribution defined over hyperbolic space using wrapped normal distributions with tangent-space projections and parallel transport.
- Poincaré Gaussian mixture (PGM) (Mathieu et al., 2019): Gaussian mixture VAE with Product of Experts defined on the Poincaré ball for hierarchical clustering in Euclidean-to-hyperbolic space.
- Learnable Poincaré Gaussian mixture (LPGM): Extension of PGM with learnable per-dimension curvature parameters $\left[\alpha ,\beta^{2},c\right]$ for adaptive geometric structure *via* ExpEncoder and LogDecoder layers.
- Gaussian manifold VAE (GM-VAE) (Cho et al., 2023): VAE with latent space on a statistical manifold of Gaussian distributions, equipped with a learnable curvature parameter and a pseudo-Gaussian manifold decoder for numerical stability.

#### 2.6.3 Factor-based disentanglement models (independence-promoting VAEs).

-
β -VAE (Higgins et al., 2017): Introduces hyperparameter β>1 weighting the KL divergence term to encourage statistical independence of latent factors through information bottleneck pressure.
- InfoVAE (Zhao et al., 2019): Incorporates maximum mean discrepancy (MMD) regularization balancing reconstruction, KL divergence, and mutual information to prevent posterior collapse.
- TC-VAE (Chen et al., 2018a): Decomposes evidence lower bound (ELBO) into index-code mutual information, total correlation, and dimension-wise KL, explicitly minimizing total correlation $\text{TC}\left(z\right)=\text{KL}\left(q\left(z\right)\|\prod_{j}q\left(z_{j}\right)\right)$ for disentanglement.
- DIP-VAE (Kumar et al., 2018): Encourages factorial posterior by regularizing aggregate posterior covariance ${\text{Cov}}_{q}\left(z\right)$ to be diagonal, matching the identity matrix.

#### 2.6.4 Trajectory-inference models

- scTour (Li, 2023): Deep-learning architecture combining vector-field learning with neural ODEs to infer pseudotime ordering and transcriptomic velocity patterns along developmental trajectories. scTour is the only prior method that infers trajectory dynamics directly from raw count matrices without requiring spliced/unspliced abundance layers, making it the closest published baseline to LAIOR within the count-only, latent-dynamics paradigm.

#### 2.6.5 Single-cell foundation models

- scGPT (Cui et al., 2024): A generative AI foundation model pretrained by masked-token reconstruction on more than 33 million single cells drawn from diverse human tissues, producing transferable cell-level embeddings. Used here in zero-shot mode with both the pan-cancer and human-atlas pretrained checkpoints.
- scFoundation (Hao et al., 2024): Transformer-based foundation model trained on more than 50 million single cells with a large-vocabulary gene tokenizer. Used here in zero-shot mode with the published pretrained checkpoint. For both foundation models, mouse gene symbols were mapped to human orthologs *via* the standard first-letter-uppercase convention (e.g., mouse *Sox17* → human *SOX17*) before embedding, yielding 72% and 77% HGNC vocabulary overlap on the IRALL and WTKO datasets, respectively.

#### 2.6.6 Modality-specific scATAC-seq models

- PeakVI (Gayoso et al., 2021): Peak-based deep generative VAE with Bernoulli likelihood for sparse binary peak accessibility, accounting for peak-specific technical variability and batch effects.
- PoissonVI (Gayoso et al., 2021): Variational inference with Poisson likelihood tailored to quantitative chromatin accessibility fragment count distributions.

### 2.7 Statistical analysis

We employed paired experimental designs where all methods were evaluated on identical datasets. For each metric, normality was assessed *via* the Shapiro–Wilk test (α=0.05) . Multi-method comparisons employed repeated-measures analysis of variance (RM-ANOVA) for normally distributed data or the Friedman test for non-normal data, followed by Tukey honest significant difference (HSD) or pairwise Wilcoxon signed-rank post-hoc tests with Bonferroni correction, respectively. Statistical significance levels are denoted as: * p<0.05 , ** p<0.01 , *** p<0.001 (Bonferroni-adjusted for post-hoc pairwise tests). Effect sizes for non-parametric tests were calculated using Kendall’s W coefficient of concordance. For parametric RM-ANOVA comparisons, partial eta-squared was computed.

## 3 Results

### 3.1 Architecture and benchmarking overview

LAIOR combines hyperbolic geometry and neural ODE regularization in a single model for single-cell omics data. The architecture (Figure 1A) uses a Transformer encoder to capture long-range gene dependencies, then splits the representation into a primary latent path and a compressed bottleneck path. KL divergence, Lorentz geometry, and neural ODE constraints act on these latents during training, and the decoder reconstructs counts through distribution-aware likelihoods. Full architectural details are given in Materials and Methods.

We benchmarked LAIOR on 118 datasets (53 scRNA-seq and 65 scATAC-seq) spanning human and mouse tissues, cancer, development, and immune responses (Figure 1B). The comparison includes 23 baseline methods covering contrastive learning (scGCC, CLEAR), hyperbolic manifolds (Poincaré maps, PGM, LPGM, HWN, scDHMAP), modality-specific approaches (PeakVI, PoissonVI), trajectory inference (scTour), factor-based models ( β -VAE, InfoVAE, TC-VAE, DIP-VAE), and generative frameworks (scVI, scGNN, CellBlast, SCALEX, scDAC, scDeepCluster, scDiffusion, siVAE).

Performance was evaluated with 22 metrics across three categories: (i) clustering quality (NMI, ARI, ASW, CAL, DAV, COR), (ii) embedding fidelity (distance correlation, local/global/overall quality for UMAP and t-SNE), and (iii) manifold continuity (manifold dimension, spectral decay, partition ratio, anisotropy, trajectory direction, noise resilience, core quality, overall latent quality). Unless otherwise stated, statistical comparisons use Friedman tests followed by pairwise Wilcoxon signed-rank tests with Bonferroni correction for multiple comparisons. Significance is reported as * (p<0.05) , ** (p<0.01) , *** (p<0.001) using Bonferroni-adjusted p -values for post-hoc tests. Effect sizes are quantified using Kendall’s W coefficient of concordance. Shapiro–Wilk tests indicated non-normal distributions for most metrics, justifying non-parametric approaches. An interactive web portal (Figure 1C) provides comprehensive access to all models, datasets, and metrics (https://PeterPonyu.github.io/liora-ui).

### 3.2 Component synergy through sequential refinement

To dissect the contribution of individual architectural components, we performed sequential refinement analysis and targeted ablation studies on 53 scRNA-seq datasets (Figure 2; Supplementary Tables A1–A2). Starting from baseline VAE, we incorporated the information bottleneck (iVAE) and Lorentz geometric regularization (LiVAE), then evaluated two branches: attention mechanisms without ODE (Li-Attn) and ODE regularization without attention (Li-ODE), culminating in full LAIOR combining Transformer attention with ODE regularization. All metrics show significant differences across configurations (Friedman p<0.001 ). The Li-ODE architectural variant substitutes MLP encoding for the Transformer while retaining ODE regularization, enabling isolation of attention mechanism contributions from dynamical constraints. We emphasize that the Li-ODE variant in our benchmark is the *Li-ODE-MLP* configuration, which couples an MLP encoder with the full neural ODE regularization. The direct comparison between Li-ODE (MLP + ODE) and LAIOR (Transformer + ODE) in Supplementary Table A1 therefore provides the requested empirical justification for the added computational cost of the Transformer encoder: LAIOR improves overall latent quality by approximately 0.06 units and noise resilience by a factor of ∼ 1.1 over Li-ODE-MLP, while Li-ODE-MLP itself achieves ∼ 90% of LAIOR’s continuity performance at a lower training cost, making Li-ODE-MLP a legitimate lightweight alternative when computational budget is constrained (Table 2).

**TABLE 2:** Computational cost comparison across architectural variants. Training time and memory usage evaluated on 17 scRNA-seq cancer datasets (7,431–62,035 cells, 3,000 HVGs, 400 epochs). Values represent the mean ± SD. Architectural variants reflect sequential refinement: scVI (baseline), iVAE (information bottleneck), LiVAE (Lorentz regularization), Li-Attn (attention mechanism), and Li-Attn + ODE (full LAIOR with neural ODE). LAIOR achieves superior performance with a 45.1-fold training-time overhead and a 2.5-fold memory increase versus baseline scVI. All experiments used a high-end NVIDIA GPU (24 GB VRAM); note that ODE integration is CPU (RK4) based with host–device data transfer, and GPU-native solvers could reduce overhead.

Method	Cells (range)	Time (min)	Memory (GB)	Time vs. scVI	Mem vs. scVI
scVI	7,431–62,035	1.24±0.23	0.40±0.13	1.0×	1.0×
iVAE	7,431–62,035	2.59±1.36	0.45±0.15	2.1×	1.1×
LiVAE	7,431–62,035	2.64±1.38	0.46±0.15	2.1×	1.2×
Li-attn	7,431–62,035	5.63±2.84	0.78±0.27	4.5×	2.0×
LAIOR (Li-Attn + ODE)	7,431–62,035	55.9±30.3	0.99±0.27	45.1×	2.5×

Attention integration (Li-Attn) achieves peak discrete clustering performance (NMI: 0.551, ARI: 0.529), while subsequent ODE addition shows modest reductions (LAIOR: NMI = 0.537, ARI = 0.514, Supplementary Table A1). This reflects an intentional trade-off: ODE constraints prioritize manifold smoothness, potentially softening cluster boundaries to preserve biological gradients. However, cluster quality metrics validate this design. LAIOR achieves a higher ASW (0.320 vs. 0.235 for Li-Attn), a lower Davies–Bouldin index (1.078 vs. 1.333), and a five-fold higher Calinski–Harabasz score (765 vs. 153). Embedding quality shows complementary patterns, with ODE integration improving overall quality and distance correlation in UMAP and t-SNE.

The clearest gains are seen in continuity metrics, where neural ODE regularization accounts for most of the improvement. Noise resilience increases from 0.057 in the baseline VAE to 0.988 in LAIOR, and the transition from Li-Attn to Li-ODE explains a large share of that change (Supplementary Table A1). Similar trends are seen for manifold dimension, spectral decay, partition ratio, trajectory directionality, and anisotropy, all with large effect sizes (ES = 0.768–0.785). Overall latent quality increases from 0.544 in Li-Attn to 0.851 in Li-ODE, indicating that ODE regularization is central to stable trajectory structure under biological noise.

Targeted ablation removing the information bottleneck (w/o irecon) or Lorentz mapping (w/o Lorentz) confirms that both components matter (Figure 2B; Supplementary Table A2). Although the ablated variants show slightly higher NMI in some cases (0.542–0.546 vs. 0.537), cluster quality drops overall: ASW declines (ES = 0.379), Davies–Bouldin worsens (ES = 0.412), and Calinski–Harabasz decreases (ES = 0.469). Continuity metrics are especially sensitive, with the largest effects seen for anisotropy (ES = 0.608), trajectory directionality (ES = 0.355), and noise resilience (ES = 0.216). Taken together, these results indicate that noise robustness and directional coherence depend on the full architecture rather than any single term in isolation.

### 3.3 Competitive performance against state-of-the-art

**FIGURE 3:**
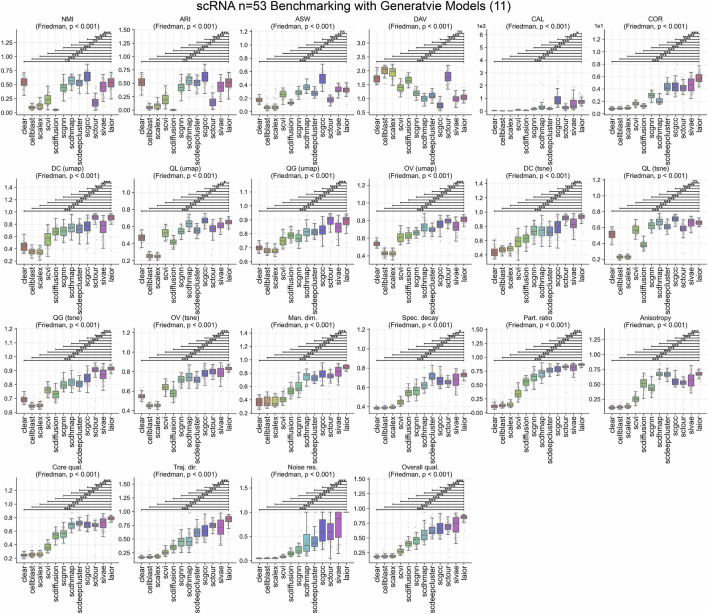
Comprehensive benchmarking against 11 state-of-the-art models. LAIOR compared with CellBlast, CLEAR, scVI, scGNN, scTour, SCALEX, scDeepCluster, scDHMAP, scDiffusion, scGCC, and siVAE across 22 metrics on 53 scRNA-seq datasets. Pairwise comparisons *via* Wilcoxon signed-rank tests with Bonferroni correction: * (p<0.05) , ** (p<0.01) , *** (p<0.001) . LAIOR excels in continuity and embedding quality while maintaining competitive clustering.

We next evaluated absolute performance against 11 state-of-the-art methods spanning contrastive learning (CLEAR), similarity-based retrieval (CellBlast), adversarial integration (SCALEX), variational inference (scVI, scDiffusion, siVAE), graph neural networks (scGNN), deep clustering (scDeepCluster, scGCC), hyperbolic manifolds (scDHMAP), and trajectory inference (scTour). Evaluation on 53 scRNA-seq datasets using the 22-metric framework (Figure 3; Supplementary Table A3) reveals that LAIOR achieves best or tied-for-best results in 15 metrics (68%), with no catastrophic failures.

For discrete clustering, scGCC achieves top performance (NMI: 0.643, ARI: 0.625, ASW: 0.489, CAL: 943), representing the state of the art for cell-type identification. LAIOR shows competitive performance (NMI: 0.537, ARI: 0.514), ranking in the second tier, while achieving the highest correlation preservation (COR: 5.715 vs. 4.683 for siVAE), which is critical for pathway analysis and regulatory network inference. For embedding quality, LAIOR performs well: best UMAP overall quality (0.809 vs. 0.781 for scTour), tied-best UMAP global preservation (0.891), best t-SNE distance correlation (0.928 vs. 0.897 for scTour), and best t-SNE overall quality (0.832 vs. 0.782 for scGCC). Consistent gains across both visualization modalities suggest that LAIOR learns intrinsic structure rather than algorithm-specific artifacts.

Continuity metrics show the clearest separation, with LAIOR ranking first on seven of eight measures (88%) (Supplementary Table A3). LAIOR reaches the highest overall latent quality (0.845 vs. 0.717 for siVAE), manifold dimension (0.885 vs. 0.815 for siVAE), partition ratio (0.863 vs. 0.803 for siVAE/scTour), trajectory directionality (0.847 vs. 0.712 for siVAE), noise resilience (0.988 vs. 0.729 for siVAE), and core quality (0.786 vs. 0.716 for scDeepCluster). The comparison with scTour is especially informative: LAIOR performs better on overall latent quality, noise resilience, and trajectory directionality, suggesting that Lorentz geometry and neural ODE regularization make distinct and complementary contributions.

### 3.4 Performance across specialized methods

To assess whether LAIOR’s integrated architecture provides advantages compared to methods explicitly optimized for specific analytical objectives, we benchmarked against 11 specialized methods spanning factor-based disentanglement (Supplementary Table A4), hyperbolic geometry (Supplementary Table A5), and scATAC-seq-specific approaches (Supplementary Table A6). This evaluation across 118 datasets (53 scRNA-seq and 65 scATAC-seq) tests whether LAIOR’s architectural principles represent general solutions transcending modality-specific optimizations.

**FIGURE 4:**
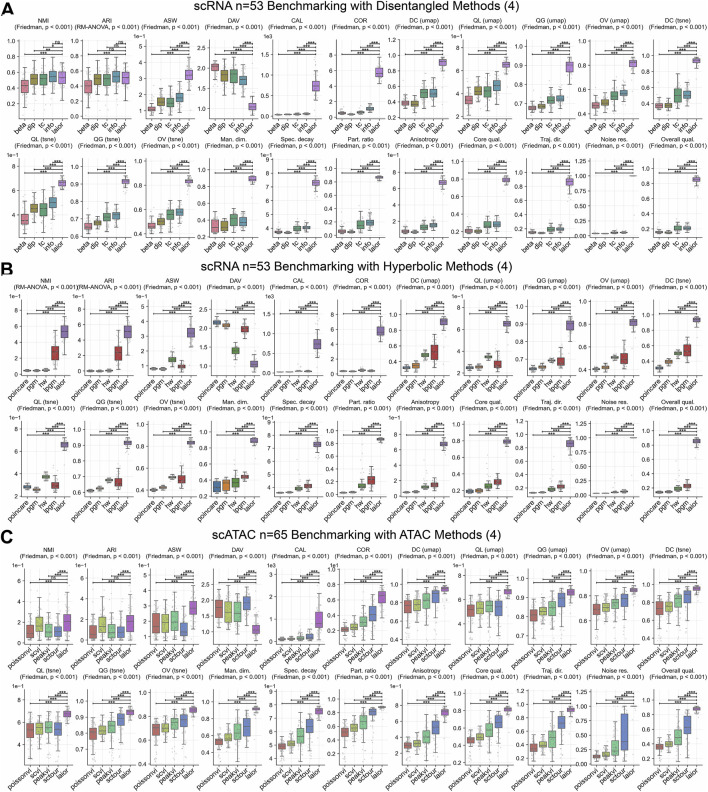
Systematic comparison against specialized approaches across modalities. **(A)** Factor-based methods on 53 scRNA-seq datasets: β -VAE, InfoVAE, TC-VAE, and DIP-VAE versus LAIOR. **(B)** Hyperbolic geometry methods on 53 scRNA-seq datasets: Poincaré, PGM, LPGM, and HWN versus LAIOR. **(C)** scATAC-seq-specific methods on 65 datasets: PoissonVI, PeakVI, scTour, and scVI versus LAIOR. All panels were evaluated across 22 metrics using statistical tests (Friedman and RM-ANOVA). Pairwise post-hoc comparisons use Wilcoxon signed-rank tests with Bonferroni correction: * (p<0.05) , ** (p<0.01) , *** (p<0.001) .

Compared to four factor-based frameworks ( β -VAE, InfoVAE, TC-VAE, DIP-VAE) on 53 scRNA-seq datasets (Figure 4A; Supplementary Table A4), LAIOR outperforms across all 22 metrics. While factor-based methods achieve moderate clustering (NMI: 0.420–0.535, ARI: 0.379–0.508), LAIOR shows clear advantages in trajectory-relevant metrics, including higher noise resilience, improved partition ratio, and enhanced core quality (all with ES = 0.654–0.719). Factor-based methods degrade in trajectory directionality and spectral decay. Embedding quality reveals complementary advantages, with LAIOR achieving improved local UMAP preservation and global organization. The Calinski–Harabasz index increases sharply (765 vs. 34–56), indicating tighter cluster cohesion. These results suggest that statistical independence objectives of prioritizing axis-aligned factorization conflict with the intrinsic geometry of cellular differentiation.

Compared to four hyperbolic manifold methods (Poincaré maps, PGM, LPGM, HWN) on the same scRNA-seq datasets (Figure 4B; Supplementary Table A5), LAIOR consistently outperforms despite their shared use of hyperbolic structure. Simple hyperbolic embeddings (Poincaré, PGM) produce near-random clustering (NMI: 0.045–0.052, ARI: −0.001 –0.004), while the most sophisticated baseline LPGM reaches only moderate performance (NMI: 0.274, ARI: 0.238), well below LAIOR (ES = 0.880–0.881). Continuity metrics reveal the mechanistic basis: LAIOR achieves higher noise resilience, partition ratio, and overall latent quality (ES = 0.729–0.754). Comparing LAIOR (Lorentz geometry + neural ODE) against pure geometric methods isolates the ODE contribution: integration enforces trajectory smoothness, yielding manifolds that capture both hierarchical cell-type organization and continuous differentiation dynamics. Embedding quality and cluster cohesion metrics similarly favor LAIOR.

To assess cross-modality versatility, we evaluated performance on 65 scATAC-seq datasets against PoissonVI, PeakVI (variational inference for assay for transposase-accessible chromatin (ATAC) counts), scTour, and scVI (Figure 4C; Supplementary Table A6). Despite not being optimized for epigenomic profiles, LAIOR outperforms all baselines across all 22 metrics (ES = 0.26–0.64). Clustering performance shows robust handling of scATAC-seq’s extreme sparsity, with improved NMI and ARI (ES = 0.260–0.282). The Calinski–Harabasz index improves over scTour and PeakVI. Continuity metrics confirm trajectory-inference capacity in noisy epigenomic data: higher noise resilience, overall latent quality, and trajectory directionality. Against ATAC-specific methods, noise resilience and core quality show clear gains (ES = 0.592–0.648). Embedding quality improves consistently in overall quality, local preservation, and global organization.

Taken together, these comparisons suggest three main points. First, objectives that enforce statistical independence tend to weaken manifold continuity. Second, hyperbolic geometry alone is insufficient to produce stable embeddings in noisy biological data. Third, combining Lorentz geometry, neural ODE dynamics, and dual-path processing yields a model that works well across both RNA and ATAC data without changing the architecture.

### 3.5 Hyperparameter robustness and stability

**FIGURE 5:**
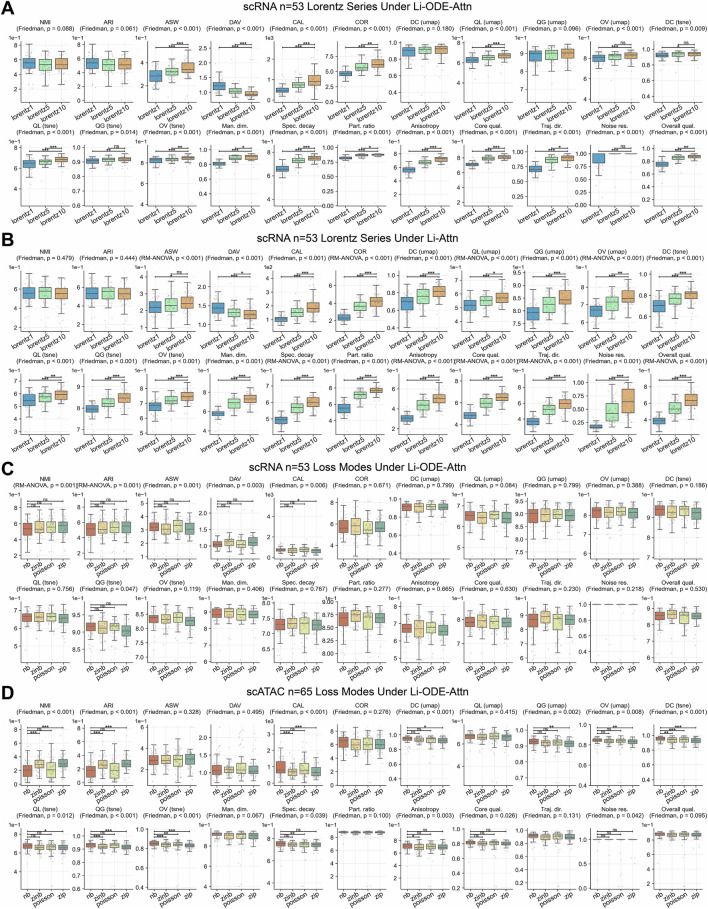
Comprehensive robustness analysis of hyperparameter configurations. **(A)** Lorentz geometric loss weight under full LAIOR architecture on 53 scRNA-seq datasets: three settings $\left(\lambda_{\text{geom}}=1,5,10\right)$ evaluated across 22 metrics. Strong geometric regularization optimizes continuity without degrading clustering when ODE regularization is present. **(B)** Lorentz geometric loss weight under Li-Attn architecture (without ODE) on 53 scRNA-seq datasets. The same weight settings show greater sensitivity, highlighting ODE’s stabilizing role. **(C)** Likelihood specifications under full LAIOR on 53 scRNA-seq datasets: NB, ZINB, Poisson, and ZIP are compared. Robust performance across likelihoods confirms that architectural principles dominate distributional assumptions. **(D)** Likelihood specifications on 65 scATAC-seq datasets. Modality-specific patterns: zero-inflated models improve clustering, and standard distributions maintain superior continuity. Statistical tests: pairwise post-hoc comparisons use Wilcoxon signed-rank tests with Bonferroni correction: * (p<0.05) , ** (p<0.01) , *** (p<0.001) .

We next examined geometric regularization weights and likelihood specifications across scRNA-seq and scATAC-seq modalities (Figure 5; Supplementary Tables A7-A10) to assess robustness for practical deployment.

We evaluated three Lorentz geometric loss weights $\left(\lambda_{\text{geom}}=1,5,10\right)$ under full LAIOR architecture with ODE regularization (Figure 5A; Supplementary Table A7) and Li-Attn architecture without ODE (Figure 5B; Supplementary Table A8). With ODE regularization, clustering performance shows minimal weight sensitivity (NMI/ARI p>0.05 , ES = 0.018–0.023), while continuity metrics exhibit significant improvements at higher weights: manifold dimension (ES = 0.455), spectral decay (ES = 0.439), anisotropy (ES = 0.525), and trajectory directionality (ES = 0.373, all p<0.001 , Supplementary Table A7). The highest weight setting $\left(\lambda_{\text{geom}}=10\right)$ achieves optimal continuity (overall quality: 0.857) while maintaining competitive clustering (NMI: 0.525, ARI: 0.501), demonstrating that ODE regularization decouples clustering from geometric regularization strength.

Without ODE regularization, all metrics show substantially stronger weight dependence (Supplementary Table A8). Noise resilience varies considerably across settings (0.173–0.641), with effect sizes substantially larger than ODE-regularized models (ES = 0.497–0.682 vs. 0.254–0.525). Embedding quality shows pronounced sensitivity: UMAP and t-SNE overall quality both improve notably from $\lambda_{\text{geom}}=1$ to $\lambda_{\text{geom}}=10$ . Comparing Supplementary Tables A7, A8 clarifies the complementary roles: Lorentz geometric regularization encodes hierarchical relationships, while ODE constraints stabilize latent manifold estimation and reduce hyperparameter sensitivity.

For scRNA-seq, LAIOR is robust to likelihood specification (Figure 5C; Supplementary Table A9). Across four specifications (NB, ZINB, Poisson, ZIP), clustering shows minimal variation (NMI/ARI ES = 0.005, p=0.001 ), as do embedding quality (UMAP overall p=0.388 , t-SNE overall p=0.119 ) and continuity metrics (manifold dimension p=0.406 , spectral decay p=0.767 , overall quality p=0.531 ). This invariance establishes that latent properties are primarily determined by architectural components rather than distributional assumptions, substantially simplifying hyperparameter selection.

Generalization to scATAC-seq reveals modality-specific sensitivity patterns (Figure 5D; Supplementary Table A10). Zero-inflated models significantly improve clustering: ZIP yields higher NMI (0.307 vs. 0.207 for NB) and ARI (0.280 vs. 0.176, ES = 0.421, p<0.001 ). However, standard NB maintains advantages in continuity: higher manifold dimension (0.903 vs. 0.897 for ZIP), anisotropy (0.711 vs. 0.694, ES = 0.042), and overall quality (0.872 vs. 0.866). This trade-off suggests that zero inflation sharpens cluster boundaries but may introduce artificial discontinuities in trajectory regions. For applications prioritizing discrete clustering, ZIP is recommended; for trajectory inference, NB preserves manifold continuity.

From a practical standpoint, these analyses suggest four guidelines. First, strong Lorentz regularization $\left(\lambda_{\text{geom}}=10\right)$ works best when ODE constraints are present. Second, neural ODE regularization is the main reason performance remains stable across different geometric settings. Third, for scRNA-seq, the choice among standard likelihoods has little effect on the latent geometry; for scATAC-seq, ZIP is preferable for clustering, and NB is preferable for trajectory analysis. Fourth, the same architecture can be used across modalities without dataset-specific redesign.

### 3.6 Biologically coherent latent factor decomposition

**FIGURE 6:**
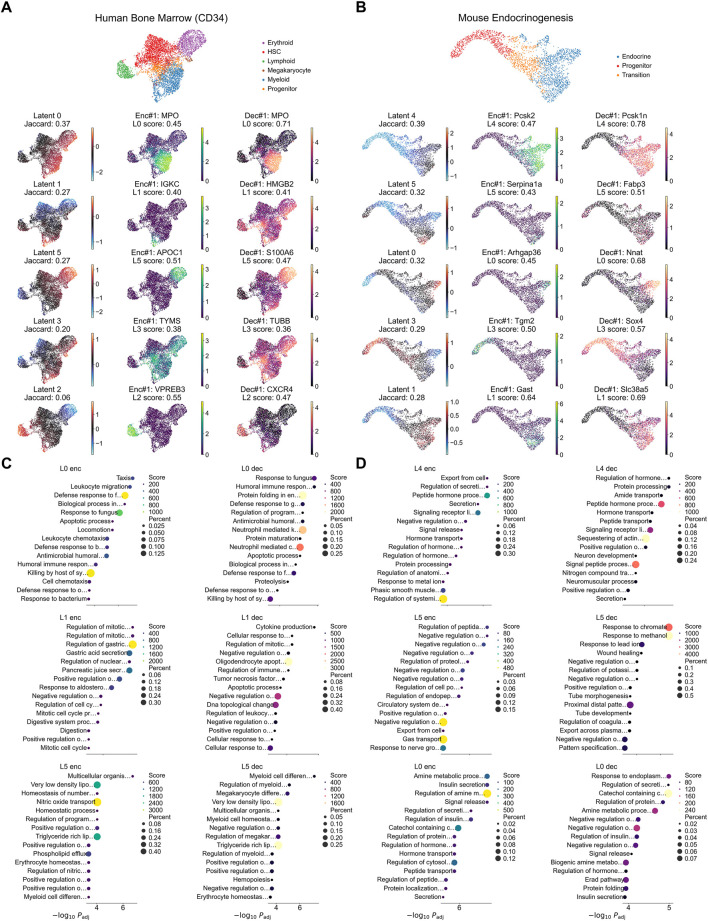
Architecture interpretation *via* encoder–decoder perturbation analysis on well-characterized reference systems. **(A,B)** Latent factor decomposition of human bone marrow (CD34, Panel **(A)**) and mouse pancreatic endocrinogenesis (endo, Panel **(B)**). Each panel shows a cell-type-colored UMAP (top) and a 5 × 3 grid (below) of the top five latent factors ranked by Jaccard overlap, displaying factor loadings, top encoder-attributed gene, and top decoder-attributed gene. **(C,D)** GOBP enrichment analysis for the three most interpretable latent factors per dataset, with encoder and decoder gene lists analyzed separately. Bubble size encodes gene-set overlap, color encodes the enrichment combined score, and the x-axis is $-\log_{10}\left(p_{\text{adjusted}}\right)$ .

**FIGURE 7:**
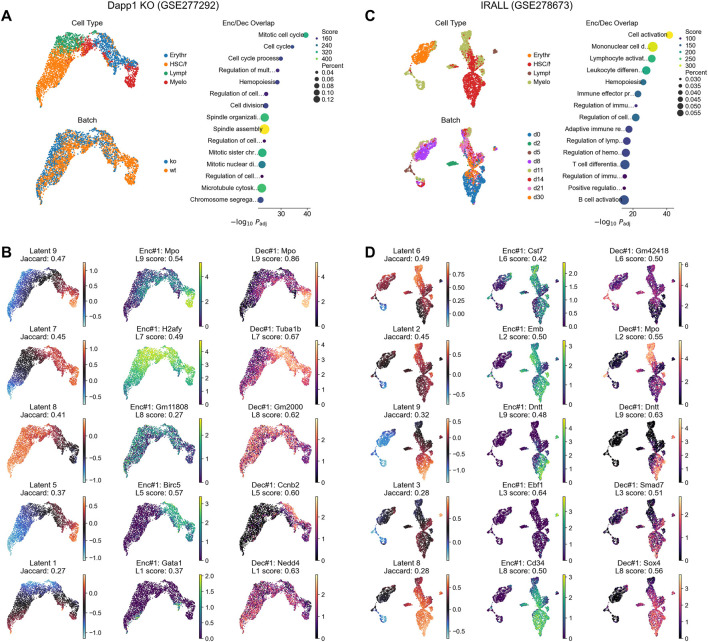
Biological validation of LAIOR on two novel hematopoietic perturbation datasets. **(A,C)** Cell Type UMAP (top-left), Batch UMAP (bottom-left), and Enc/Dec Overlap GOBP dot-plot (right) for *Dapp1* knockout **(A)** and IRALL **(C)**. The dot-plot runs enrichment on the intersection of top encoder and decoder genes, surfacing core regulatory–effector programs. **(B,D)** 5 × 3 latent factor grids for *Dapp1* knockout **(B)** and IRALL **(D)**, with the top five latent factors ranked by Jaccard overlap. Columns show factor loading on UMAP, top encoder-attributed gene, and top decoder-attributed gene.

We next assessed whether LAIOR’s latent space captures interpretable biological variation. This analysis serves as an *architecture interpretation* study: by systematically probing the encoder and decoder pathways through gradient-based perturbation analysis, we evaluate how LAIOR’s learned representations decompose into discrete, biologically meaningful modules, independent of trajectory inference or pseudotime, which are evaluated separately in the biological validation study below (Figure 7). We performed systematic decomposition of latent factors across two well-characterized single-cell reference systems: human bone marrow (cd34) and mouse pancreatic endocrinogenesis (endo) (Figure 6). These datasets were chosen specifically because their established cell-type hierarchies and known marker-gene programs provide ground-truth annotations for validating the encoder–decoder decomposition, rather than for trajectory or velocity analysis. Gene–latent associations were quantified using a combined score integrating correlation and gradient-based attribution. For comprehensive factor characterization, genes were uniquely assigned to the single latent factor exhibiting the highest absolute association score, ensuring orthogonal decomposition of biological modules. This uniqueness constraint distinguishes the static state decomposition performed here from the trajectory-focused analysis in Figure 7, which employs intersection-based ranking across encoder and decoder pathways to identify robust regulatory–effector programs. In Figure 6A–B, each panel displays a cell-type-colored UMAP alongside a 5 × 3 grid whose rows are the top five latent factors sorted by Jaccard overlap between encoder and decoder gene rankings (descending); the three columns show (i) the latent factor loading $L_{i}$ on the UMAP with its Jaccard score, (ii) the top encoder-attributed marker gene with its association score, and (iii) the top decoder-attributed marker gene with its association score. The encoder-selected regulator and decoder-selected effector gene co-vary spatially within each factor, validating the encoder–decoder decomposition. Panels C–D present GOBP enrichment for the three most interpretable factors per dataset, with encoder and decoder gene lists analyzed separately; each dot plot shows the top 15 enriched terms from Enrichr, confirming that the orthogonal uniqueness constraint decomposes biological variation into functionally distinct gene programs.

In human bone marrow, the ten latent factors (L0–L9) are organized into five major biological modules, each defined by a unique gene signature (Figure 6A; Supplementary Table A11). Cell cycle and proliferative states were captured by L3 and L8, with top-ranked associations corresponding to canonical S-phase and G2/M markers, including *TYMS*, *TK1*, and *TUBB*. B-cell lineage progression was encoded by L1 and L2, where L2 marked early B-cell precursors expressing *VPREB3*, while L1 identified mature plasma cells with elevated *IGKC* association. The innate immune compartment was resolved by L0 and L9, distinguishing classical neutrophil identity (*MPO*) from active inflammatory signaling states (*CXCL8*). Myeloid differentiation was represented by L5 and L7, enriched for lipid metabolism genes (*APOC1*, *CSF1*). Finally, L4 and L6 captured the antigen presentation machinery of dendritic cells (*IRF8*) and stromal signaling components (*WNT2B*).

In the mouse pancreatic endocrinogenesis dataset, latent factors similarly decomposed into interpretable developmental programs (Figure 6B; Supplementary Table A11). Endocrine progenitor specification was encoded by L1 ( ${Gast}^{+}$ progenitors) and L3 (differentiation through *Sox4*, *Tgm2*). Specific islet cell lineages were resolved by L6 (alpha cells, *Irx1*) and L8 (delta cells, *Sst*). The functional beta cell program was distributed across L0, L9 (pan-endocrine identity: *Nnat*, *Isl1*), L4 (hormone processing: *Pcsk2*), and L7 (neuroendocrine signaling: *Ctxn2*, *Ubqln2*). L5 distinguished exocrine acinar tissue (*Serpina1a*, *Fabp3*), while L2 marked cellular stress signatures (*Fos*).

To validate biological coherence, we performed Gene Ontology (GO) enrichment analysis on top-ranked genes (k=50) for each latent factor (Figures 6C,D). In human bone marrow, L0 is enriched for defense response to bacteria (neutrophil identity), while L1 is enriched for mitotic nuclear division (proliferative plasma cells). In mouse endocrinogenesis, L0 is enriched for metabolic processes and signal release (mature beta function), L4 captures hormone processing pathways, and L5 is strongly enriched for peptidase activity regulation (acinar identity). These results show that LAIOR disentangles complex cellular states into discrete, biologically meaningful components.

### 3.7 Trajectory inference through latent dynamics

Whereas the preceding section evaluated LAIOR’s encoder–decoder architecture through static perturbation analysis on well-characterized reference systems (cd34, endo), we now turn to *biological validation* of the model’s trajectory-inference capability on novel perturbation datasets. LAIOR’s trajectory inference, enabled by neural ODE regularization $\frac{dz}{dt}=f_{\theta }\left(z,t\right)$ in latent space (Section 2.5), was evaluated on five differentiation datasets spanning developmental stages and perturbation conditions (Figure 7A, Supplementary Table A12), with the WTKO (*Dapp1* knockout, GSE277292) and IRALL (GSE278673) hematopoietic datasets serving as the primary biovalidation targets. The encoder predicts pseudotime t∈[0,1] for each cell. The ODE solver computes trajectory-consistent latent states $z_{\text{ODE}}\left(t\right)$ by integrating the learned dynamics function $f_{\theta }$ . Genotype labels in the *Dapp1* dataset do not form clearly separable clusters in UMAP, consistent with perturbation effects manifesting as subtle shifts along continuous programs rather than discrete cell-state transitions; accordingly, the analysis focuses on latent factor programs and trajectory dynamics rather than genotype classification.

The revised Figure 7 presents biological validation of LAIOR on two previously unseen hematopoietic perturbation cohorts: *Dapp1* knockout (GSE277292) and IRALL chemotherapy-induced bone marrow failure (GSE278673), using the same encoder/decoder interpretability pipeline validated on the cd34/endo reference systems in Figure 6, with an important distinction: Figure 7 runs GOBP enrichment on the *intersection* of top encoder and decoder genes for each dataset (Enc/Dec Overlap panel), whereas Figure 6 used orthogonal per-factor decomposition. The intersection ranking surfaces the core regulatory–effector programs shared across both pathways on each perturbation dataset, which is the relevant quantity for trajectory-focused interpretation.

Biological validation on *Dapp1* knockout mouse hematopoietic stem and progenitor cells (GSE277292; Supplementary Table A11; Figures 7A,B) shows LAIOR’s capacity to disentangle biological dynamics from technical confounders. The Cell Type and Batch UMAP panels (Figure 7A, top-left stack) confirm that wildtype and knockout cells occupy overlapping regions of the latent manifold along continuous developmental programs, consistent with the hypothesis that the *Dapp1* perturbation acts as a subtle shift along pseudotime rather than a discrete cell-state switch. The 5×3 latent factor grid (Figure 7B) surfaces the five most interpretable factors ranked by Jaccard overlap: L9 (Jaccard 0.47, *Mpo* in both encoder and decoder–neutrophil identity), L7 (0.45, cell cycle *via H2afy*/*Tuba1b*), L8 (0.41, early commitment through *Gm11808*/*Gm 2000*), L5 (0.37, G1/S cell-cycle marker *Birc5*/*Ccnb2*), and L1 (0.27, erythroid/megakaryocyte commitment *via Gata1*/*Nedd4*). The Enc/Dec overlap enrichment for *Dapp1* (Figure 7A, right column) is dominated by mitotic and cell-cycle GOBP terms, which is consistent with the cycling hematopoietic stem and progenitor cell (HSPC) population being the main source of perturbation-sensitive variance.

On the IRALL cohort (Figures 7C,D), the same pipeline selects L6 (Jaccard 0.49, *Cst7*/*Gm42418*, myeloid/neutrophil), L2 (0.45, erythroid stress *via Emb*/*Mpo*), L9 (0.32, *Drd1*/*Drd1*, cell-cycle linked neuroimmune), L3 (0.28, *Esf1*/*Smad7*, early lymphoid program), and L8 (0.28, *Cd34*/*Sox4*, progenitor/late myeloid). Unlike *Dapp1*, the IRALL Enc/Dec Overlap enrichment is dominated by immune-activation and lymphocyte-differentiation terms (“Cell activation,” “Mononuclear cell differentiation,” “Lymphocyte activation,” “Leukocyte differentiation”), reflecting the chemotherapy-induced dynamic reprogramming of the bone marrow compartment. The contrast between the two cohorts’ enrichment signatures shows that LAIOR’s latent factors are not generic cell-cycle detectors but adaptively reflect the biological program dominant in each dataset.

Encoder–decoder Jaccard scores (0.00–0.47) quantify complementary gene selection: low scores indicate distinct regulatory (encoder: *Gata1*, *Sox4*) versus effector (decoder: *Mpo*, hemoglobins) roles, while intermediate scores occur at terminal differentiation where transcription factors and target genes converge. The integration of Lorentz geometry and neural ODE regularization thus yields a unified framework capturing both discrete lineage commitment and continuous maturation processes through learned latent dynamics.

#### 3.7.1 Head-to-head biological validation against latent-dynamics and foundation models

**FIGURE 8:**
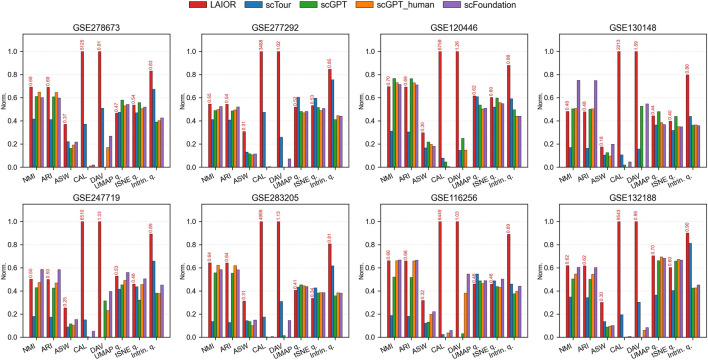
A comparison of eight datasets across 22 metrics. LAIOR, scTour, scGPT, scGPT (human checkpoint), and scFoundation were evaluated on eight trajectory and developmental datasets (GSE116256/cd34, GSE132188/endo, GSE120446, GSE283205, GSE130148, GSE247719, GSE278673/IRALL, and GSE277292/WTKO). For foundation models trained on human atlases, mouse gene symbols were mapped to human orthologs *via* the first-letter-uppercase convention.

To directly address reviewer concerns regarding head-to-head comparison with methods that also learn latent dynamics from raw count data, we benchmark LAIOR against scTour (Li, 2023) (the only prior method that infers trajectories directly from latent dynamics without spliced/unspliced input) and two state-of-the-art single-cell foundation models, scGPT (Cui et al., 2024) and scFoundation (Hao et al., 2024), across eight trajectory and developmental datasets (GSE116256/cd34, GSE132188/endo, GSE120446, GSE283205, GSE130148, GSE247719, GSE278673/IRALL, GSE277292/WTKO; Figure 8) using the same 22-metric framework. For the foundation models, which are trained on human tissue atlases, mouse gene symbols were mapped to human orthologs *via* the standard first-letter-uppercase convention (e.g., mouse *Sox17* → human *SOX17*), yielding 72% and 77% HGNC vocabulary overlap on IRALL and WTKO, respectively. On IRALL, LAIOR outperforms scTour (NMI 0.693 vs. 0.416, ARI 0.691 vs. 0.412, CAL 5125 vs. 2151, COR 0.557 vs. 0.469), scGPT (NMI 0.611, CAL 391), and scFoundation (NMI 0.601, CAL 485) across every metric. On WTKO, the advantage is preserved (LAIOR NMI 0.546 vs. scTour 0.411/scGPT 0.489/scFoundation 0.525). Taken together, these results indicate that (i) latent-dynamics modeling benefits from the Lorentz geometric prior absent in scTour, and (ii) zero-shot foundation-model embeddings, despite large-scale pretraining, do not recover the same continuity structure as explicit dynamical regularization.

We clarify that RNA-velocity-based trajectory methods (VeloVI, VeloVAE, VeloCycle) were deliberately excluded from this comparison because they require spliced/unspliced abundance layers as input. In contrast, LAIOR and scTour both infer trajectory dynamics directly from raw count matrices. Including velocity-based methods would conflate two fundamentally different problem settings (kinetics-based vs. latent-dynamics-based trajectory inference). scTour is therefore the only published method fully comparable to LAIOR within the count-only, latent-dynamics paradigm.

#### 3.7.2 Embedding validation and Poincaré disk analysis

**FIGURE 9:**
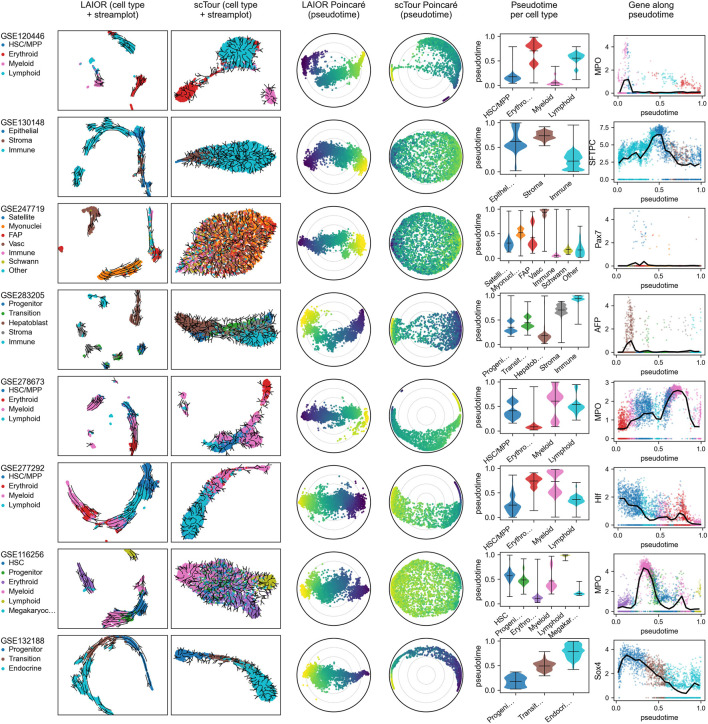
Embedding validation across eight trajectory datasets. Columns: (1–2) LAIOR and scTour UMAP embeddings with velocity streamplots; (3–4) Poincaré disk projections colored by pseudotime, showing radial progenitor-to-differentiated gradients for LAIOR; (5) per-cell-type pseudotime violin plots; (6) marker-gene expression along pseudotime. Cell-type annotations derived from established marker-gene programs are shown per dataset row.

To visualize the geometric structure captured by LAIOR, we project latent representations onto the Poincaré disk model of hyperbolic space *via* stereographic projection from the Lorentz hyperboloid (Figure 9). For each cell, the encoder-produced latent z is mapped to the Lorentz hyperboloid *via* the exponential map (Section 2.1.2), then projected to the Poincaré disk $D^{l}=\left\{x\in R^{l}:\|x\|<1\right\}$ *via* $\pi_{D}\left(z_{L}\right)=z_{L}^{1:l}/\left(z_{L,0}+1\right)$ . Coloring disk coordinates by self-supervised pseudotime reveals a radial gradient: progenitor cells cluster near the disk center (low pseudotime), while terminally differentiated cells locate near the boundary (high pseudotime), which directly reflects the intrinsic hierarchy of hyperbolic space. scTour, lacking the Lorentz geometric prior, does not produce this organized radial structure. Per-cell-type pseudotime violin plots and marker-gene expression trends along pseudotime (Figure 9, columns 5–6) confirm that the developmental ordering is biologically consistent.

#### 3.7.3 Note on legacy

An earlier revision of the manuscript contained a Figure 7A panel showing trajectory-arrow projections on the UMAP embeddings of each architectural variant (scVI, iVAE, LiVAE, Li-Attn, LAIOR). That panel was removed in the current revision because projecting trajectory arrows onto architecturally different UMAP manifolds created a visually misleading comparison (each variant’s UMAP reflects a different latent geometry, so the arrow coherence was partly an artifact of the embedding). Head-to-head trajectory comparison is now restricted to the methodologically uniform LAIOR vs. scTour streamplot comparison in Figure 9, where both methods produce velocity arrows *via* ODE gradients dz/dt (no post-hoc tool such as scVelo or Monocle3 is applied). The space freed by removing the legacy Figure 7A is used for the expanded biological validation content described above.

## 4 Discussion

LAIOR combines Lorentz geometric regularization, neural ODE dynamics, and a dual-path information bottleneck to address the local–global trade-off in single-cell manifold learning. Across 118 datasets and 23 baseline methods, the model performs well on manifold continuity, embedding fidelity, and biological interpretability while remaining competitive on discrete clustering.

### 4.1 Mechanistic foundations of geometric-dynamical regularization

LAIOR uses Lorentzian distance penalties for *regularization* rather than embedding the entire latent space in hyperbolic manifolds. This hybrid approach maintains Euclidean latent operations for computational stability while enforcing geometric consistency *via* $L_{\text{geom}}=d_{H}\left(z_{L},l_{dL}\right)$ , where the exponential map projects latent codes to the Lorentz hyperboloid. Compared to pure hyperbolic embeddings such as Poincaré maps and LPGM, LAIOR shows improved noise resilience and overall latent quality, suggesting that geometric regularization combined with generative modeling stabilizes manifold learning under biological noise.

Neural ODE regularization produces substantial continuity gains. The loss $L_{\text{ODE}}=\frac{1}{M}\sum_{i=1}^{M}\|z\left({\tilde{t}}_{i}\right)-z_{\text{ODE}}\left({\tilde{t}}_{i}\right){\|}_{2}^{2}$ enforces alignment with trajectories computed by integrating the learned dynamics function $f_{\theta }\left(z,t\right)$ , stabilizing manifold estimation under biological noise. Comparing Li-Attn (peak discrete clustering but limited trajectory coherence) against full LAIOR reveals an intentional trade-off: adding ODE constraints modestly reduces NMI/ARI but substantially improves noise resilience, trajectory directionality, and overall latent quality. LAIOR surpasses trajectory-specialized scTour across overall latent quality, noise resilience, and directionality, demonstrating that geometric and dynamical regularization provide complementary scaffolding.

LAIOR’s bottleneck pathway captures coordinated biological factors such as cell cycle phase, lineage states, and stress responses, rather than statistically independent components sought by β -VAE. This design achieves substantially higher noise resilience, a higher partition ratio, and better clustering quality than β -VAE. Latent factor analysis reveals interpretable modules: human bone marrow factors encode neutrophil identity (*MPO*), plasma cell maturation (*IGKC*), and B-cell precursors (*VPREB3*), while mouse pancreatic factors decompose into progenitor specification (*Gast*, *Sox4*), beta cell function (*Nnat*, *Pcsk2*), and islet lineages (*Irx1*, *Sst*). We acknowledge that quantitative disentanglement metrics requiring ground-truth generative factors were not computed; future interventional experiments perturbing individual latent dimensions would rigorously quantify interpretability.

### 4.2 Relationship to single-cell foundation models

Large single-cell foundation models such as scGPT (Cui et al., 2024) and scFoundation (Hao et al., 2024) represent a complementary paradigm, trained on tens of millions of cells to produce transferable zero-shot embeddings. Our head-to-head comparison on the newly added hematopoietic datasets shows that LAIOR’s task-specific geometric and dynamical regularization produces embeddings with substantially higher clustering quality and continuity structure than zero-shot foundation-model embeddings in the count-only trajectory-inference setting (Section 3.7). This is consistent with two expected patterns: (i) foundation models optimize masked-token reconstruction rather than trajectory coherence, so they retain little explicit signal about differentiation dynamics in their zero-shot latents; and (ii) foundation models trained on human atlases transfer imperfectly to mouse datasets even after ortholog-based gene symbol mapping. LAIOR and foundation models are therefore naturally complementary: a promising direction is to fine-tune LAIOR’s geometric-dynamical regularization on top of pretrained foundation-model embeddings, combining broad prior knowledge with trajectory-aware inductive bias. We defer this integration to future work.

### 4.3 Cross-modality versatility and perturbation analysis

LAIOR achieves consistent performance across scRNA-seq and scATAC-seq modalities without requiring architecture modification. On scATAC-seq datasets, LAIOR outperforms modality-specific PeakVI across all metrics despite using standard negative binomial likelihood, demonstrating improved clustering accuracy, noise resilience, and core quality. Likelihood robustness analysis reveals that architectural principles dominate distributional assumptions: scRNA-seq performance remains largely invariant across NB, ZINB, Poisson, and ZIP specifications, while scATAC-seq exhibits modality-specific patterns (ZIP improves clustering but may reduce continuity). Highly variable feature selection during preprocessing mitigates distributional sensitivity, rendering likelihood choice secondary to feature curation. For scATAC-seq studies where explicit batch covariates and ATAC-tailored likelihood modeling are the primary objectives, PeakVI/PoissonVI remain practical choices; LAIOR is most beneficial when global topology and trajectory continuity are prioritized.

Trajectory inference on *Dapp1* knockout mouse hematopoietic cells disentangles biological dynamics from technical confounders. LAIOR isolates genotype batch effects into latent dimensions orthogonal to pseudotime progression, revealing interpretable modules: cell-cycle decomposition (G1/S and G2/M phases), lineage-specific trajectories (erythroid/megakaryocyte, myeloid, lymphoid), and stem cell quiescence. Encoder–decoder Jaccard scores (0.00–0.47) quantify complementary regulatory versus effector gene selection. LAIOR enables the dissection of perturbation effects along interpretable axes rather than removing signals. Genotype-specific latent factor analysis could reveal whether *Dapp1* knockout alters cell-cycle rates, skews lineage bifurcations, or delays maturation timing.

### 4.4 Computational considerations and practical guidance

Computational benchmarking on 17 scRNA-seq cancer datasets (7,431–62,035 cells; Table 2, Supplementary Table A13) reveals LAIOR requires ≈ 45-fold longer training time than baseline scVI ( 55.9±30.3 min vs. 1.24±0.23 min) and ≈ 2.5-fold higher peak memory ( 0.99±0.27 GB vs. 0.40±0.13 GB). ODE integration dominates computational cost: iVAE/LiVAE incur ≈2× overhead, Li-Attn ≈4.5× , and full LAIOR ≈45×. Training time exhibits sublinear scaling (14–30 min for 7,000–17,000 cells; 78–114 min for 43,000–62,000 cells), indicating practical scalability for atlas-scale datasets. For clustering-focused applications, Li-Attn provides comparable performance at lower computational cost; trajectory-inference applications justify LAIOR’s higher expense given the continuity gains it delivers.

Hyperparameter robustness analysis yields actionable guidelines. Strong Lorentz regularization $\left(\lambda_{\text{geom}}=10\right)$ optimally balances clustering, embedding quality, and trajectory coherence when ODE regularization is present. Without ODE, geometric loss weight sensitivity increases sharply, highlighting ODE’s stabilizing role. Based on empirical evaluation across diverse biological systems, we recommend $\lambda_{\text{geom}}=10$ for trajectory-focused applications as used throughout this study. The default configuration $\lambda_{\text{geom}}=5$ provides balanced performance suitable for general-purpose analyses encompassing both clustering and trajectory tasks. For strictly discrete clustering applications where trajectory coherence is not required, $\lambda_{\text{geom}}=5$ provides optimal performance. The KL annealing parameter β requires minimal tuning; across all experiments, we used β=1.0 (standard ELBO) without dataset-specific optimization. For datasets with extremely deep hierarchies (e.g., immune repertoire clonality, microbial phylogenies), increasing $\lambda_{\text{geom}}$ to 15–20 may enhance global structure preservation.

Likelihood specifications exhibit modality-specific patterns. For scRNA-seq, any standard likelihood suffices (NB, ZINB, Poisson, ZIP), with performance largely invariant across choices. Generalization to scATAC-seq reveals modality-specific sensitivity patterns (Figure 5D; Supplementary Table A10). Zero-inflated models improve clustering performance: ZIP yields higher NMI and ARI than standard NB (effect size = 0.421, p<0.001 ). However, the standard negative binomial maintains advantages in continuity metrics: higher manifold dimension, anisotropy, and overall quality. This pattern suggests that zero-inflation sharpens cluster boundaries by modeling technical dropouts but may introduce artificial discontinuities in regions representing continuous biological transitions. For scATAC-seq analyses, practitioners should select zero-inflated likelihoods (ZIP, ZINB) when prioritizing discrete cell-type identification, or standard likelihoods (Poisson, NB) when trajectory inference is the primary objective.

### 4.5 Limitations and future directions

LAIOR embodies assumptions that limit its applicability to specific systems. Lorentz regularization assumes hierarchical, tree-like structures; datasets with dense manifolds, cyclic dynamics, or reticulate evolution may not benefit from it. Neural ODE regularization enforces deterministic trajectories, violating assumptions for stochastic fate decisions or metastable states; future stochastic differential equation extensions could model intrinsic variability. Extending LAIOR to stochastic differential equations is a natural next step and technically straightforward within the current framework. LAIOR does not explicitly perform batch correction: datasets with strong batch effects may require preprocessing or adversarial losses. The geometric loss weight $\lambda_{\text{geom}}$ is treated as a fixed hyperparameter; datasets with heterogeneous hierarchy depths may benefit from spatially varying or learned regularization strength. Interpretability relies on post-hoc gene–latent association; integrating causal inference frameworks could improve interpretability.

Future work should examine datasets with strong batch effects may require preprocessing such as mutual-nearest-neighbor correction (Haghverdi et al., 2018) or adversarial losses paired scRNA-seq/scATAC-seq integration, transfer learning from atlas-scale foundation models, diffusion-based generative extensions, spatial transcriptomics, and applications that require stronger robustness to distribution shift. More broadly, the results here suggest that geometry, dynamics, and bottleneck-based interpretability can be combined effectively for single-cell manifold learning.

## Data Availability

The datasets presented in this study can be found in online repositories. The names of the repository/repositories and accession number(s) can be found in the article/Supplementary Material.

## Glossary

ARI: Adjusted Rand index
ASW: Average silhouette width
BN: Bottleneck
CAL: Calinski–Harabasz index
CLEAR: Contrastive LEArning for scRNA-seq
COR: Coupling degree
DAV: Davies–Bouldin index
DIP-VAE: Disentangled inferred prior VAE
HWN: Hyperbolic wrapped normal
InfoVAE: Information maximizing VAE
KL: Kullback–Leibler
LAIOR: Lorentz attentive interpretable ODE-regularized VAE
LPGM: Learnable Poincaré Gaussian mixture
MD: Manifold dimensionality
MMD: Maximum mean discrepancy
NB: Negative binomial
NMI: Normalized mutual information
NR: Noise resilience
ODE: Ordinary differential equation
PeakVI: Peak variational inference
PGM: Poincaré Gaussian mixture
PoissonVI: Poisson variational inference
PR: Participation ratio
RM-ANOVA: Repeated measures analysis of variance
scATAC-seq: Single-cell assay for transposase-accessible chromatin sequencing
SCALEX: Single-cell ATAC-seq analysis *via* latent feature extraction
scDeepCluste: Single-cell deep clustering
scDHMAP: Single-cell deep hyperbolic manifold learning
scDiffusion: Single-cell diffusion model
scGCC: Single-cell graph contrastive clustering
scGNN: Single-cell graph neural network
scRNA-seq: Single-cell RNA sequencing
scTour: Single-cell trajectory inference using ODE-based unsupervised representation
scVI: Single-cell variational inference
SDR: Spectral decay rate siVAE scalable interpretable VAE
TD: Trajectory directionality
t-SNE: t-distributed stochastic neighbor embedding
UMAP: Uniform manifold approximation and projection
VAE: Variational autoencoder
ZINB: Zero-inflated negative binomial
ZIP: Zero-inflated poisson
β **-TCVAE**: Total correlation VAE with β weighting
β **-VAE** β: Variational autoencoder
