# Supplementary material for: LAIOR: a hyperbolic neural ODE variational framework for interpretable single-cell manifold learning and trajectory inference
Source: Front Genet. 2026 Jun 8;17:1838613. doi: 10.3389/fgene.2026.1838613 (PMC13283489; doi:10.3389/fgene.2026.1838613)
Supplement: Supplementary file 1 [file Supplementaryfile1.pdf]

Supplementary Tables A1–A13

LAIOR: A Hyperbolic Neural-ODE Variational Framework for Interpretable Single-Cell Manifold Learning and Trajectory Inference

Zeyu Fu, Jiawei Fu, Keyang Zhang, Tianfei Ran, Chunlin Chen

This supplementary file contains the thirteen result/statistical data-point tables (Supplementary Tables A1–A13) cited in the main article. The dataset summary and sample-level GEO mapping for the 53 scRNA-seq and 65 scATAC-seq samples (118 total) are provided in the separate file `Supplementary_datatables.pdf`.

Supplementary Table A1. Architectural refinements of the LAIOR model evaluated across 53 scRNA-seq datasets. This table tracks sequential model improvements from the VAE baseline through Identifiable VAE (iVAE), Lorentz hyperbolic mapping (LiVAE), attention integration (Li-Attn), and neural ODE regularization (Li-ODE), culminating in the full LAIOR model. Attention mechanisms show peak performance in discrete clustering (NMI: 0.551, ARI: 0.529), while the integration of neural ODEs significantly optimizes manifold coherence and embedding quality. Hyperbolic geometry and ODE regularization are critical for latent space continuity, with noise resilience showing a 17.3-fold improvement from the VAE baseline to the final model. All 22 metrics demonstrate statistical significance via Friedman tests ($p < 0.001$). Values represent mean performance across all datasets; bold entries indicate the optimal performance for each metric.

**Metric**	**VAE**	**iVAE**	**LiVAE**	**Li-Attn**	**Li-ODE**	**LAIOR**	**Test**	**P-value**	**ES**
Clustering and Coupling									
NMI	0.5279	0.5501	0.5401	**0.5511**	0.5135	0.5365	Friedman	<0.001	0.133
ARI	0.5049	0.5280	0.5174	**0.5291**	0.4897	0.5137	Friedman	<0.001	0.133
ASW	0.1793	0.2189	0.2293	0.2354	0.2960	**0.3199**	Friedman	<0.001	0.633
DAV	1.6976	1.4813	1.3787	1.3331	1.1323	**1.0781**	Friedman	<0.001	0.629
CAL	55.9399	102.7830	154.3786	152.9733	727.9169	**765.2788**	Friedman	<0.001	0.766
COR	1.1373	2.3041	3.4835	3.5707	**5.8548**	5.7153	Friedman	<0.001	0.744
Embedding Quality: UMAP									
DC (umap)	0.5150	0.6917	0.7660	0.7546	**0.9144**	0.8890	Friedman	<0.001	0.650
Q_{local} (umap)	0.4663	0.5149	0.5412	0.5469	**0.6480**	0.6458	Friedman	<0.001	0.667
Q_{global} (umap)	0.7274	0.7961	0.8311	0.8250	**0.9063**	0.8910	Friedman	<0.001	0.674
Overall (umap)	0.5696	0.6676	0.7128	0.7088	**0.8229**	0.8086	Friedman	<0.001	0.670
Embedding Quality: t-SNE									
DC (tsne)	0.5073	0.6937	0.7536	0.7636	**0.9330**	0.9277	Friedman	<0.001	0.703
Q_{local} (tsne)	0.4929	0.5420	0.5592	0.5682	0.6545	**0.6581**	Friedman	<0.001	0.660
Q_{global} (tsne)	0.7204	0.7936	0.8251	0.8264	**0.9181**	0.9111	Friedman	<0.001	0.734
Overall (tsne)	0.5735	0.6764	0.7126	0.7194	**0.8352**	0.8323	Friedman	<0.001	0.722
Continuity Quality: Latent Space									
Manifold Dim.	0.3844	0.5716	0.6738	0.6824	0.8834	**0.8850**	Friedman	<0.001	0.768
Spectral Decay	0.4071	0.4897	0.5604	0.5672	**0.7261**	0.7225	Friedman	<0.001	0.776
Partition Ratio	0.2030	0.5260	0.6955	0.7137	**0.8663**	0.8626	Friedman	<0.001	0.777
Anisotropy	0.1590	0.2939	0.4167	0.4346	**0.6725**	0.6719	Friedman	<0.001	0.778
Trajectory Dir.	0.1994	0.3570	0.5065	0.5220	**0.8709**	0.8473	Friedman	<0.001	0.777
Noise Resilience	0.0570	0.1560	0.4092	0.4395	0.9790	**0.9876**	Friedman	<0.001	0.785
Core Quality	0.2884	0.4703	0.5866	0.5995	**0.7871**	0.7855	Friedman	<0.001	0.776
Overall Quality	0.2154	0.3734	0.5271	0.5442	**0.8506**	0.8445	Friedman	<0.001	0.777

Supplementary Table A2. Ablation study of core LAIOR components across 53 scRNA-seq datasets. This analysis examines the necessity of the Information Bottleneck reconstruction (irecon) and Lorentz hyperbolic mapping. Results demonstrate that while ablated variants may occasionally show higher scores in discrete clustering (e.g., NMI), the full LAIOR model is essential for maintaining superior ASW, DAV, and CAL scores. Local embedding quality and global continuity metrics are highly sensitive to the removal of these components, with anisotropy and trajectory direction showing the most significant dependence on the full integrated architecture. The synergy between components ensures noise resilience and overall manifold quality. Bolded values indicate the highest mean performance for each metric across the 53 datasets.

Metric	w/o irecon	w/o Lorentz	Full LAIOR	Test	P-value	ES
Clustering and Coupling						
NMI	0.5419	**0.5463**	0.5365	Friedman	0.0283	0.033
ARI	0.5195	**0.5240**	0.5137	Friedman	0.0283	0.033
ASW	0.2639	0.2835	**0.3199**	Friedman	<0.001	0.379
DAV	1.3427	1.2499	**1.0781**	Friedman	<0.001	0.412
CAL	439.0753	492.6384	**765.2788**	Friedman	<0.001	0.469
COR	4.6790	4.5782	**5.7153**	RM-ANOVA	<0.001	0.372
Embedding Quality: UMAP						
DC (umap)	0.8872	0.8826	**0.8890**	Friedman	0.4443	0.000
Q_{local} (umap)	0.6161	0.6187	**0.6458**	Friedman	<0.001	0.133
Q_{global} (umap)	0.8834	0.8820	**0.8910**	Friedman	0.0813	0.019
Overall (umap)	0.7956	0.7945	**0.8086**	Friedman	0.0007	0.081
Embedding Quality: t-SNE						
DC (tsne)	0.9271	0.9220	**0.9277**	Friedman	0.0462	0.027
Q_{local} (tsne)	0.6328	0.6400	**0.6581**	Friedman	0.0048	0.056
Q_{global} (tsne)	0.9052	0.9013	**0.9111**	Friedman	0.0283	0.033
Overall (tsne)	0.8217	0.8211	**0.8323**	Friedman	0.0149	0.041
Continuity Quality: Latent Space						
Manifold Dim.	0.7916	0.7956	**0.8850**	Friedman	<0.001	0.482
Spectral Decay	0.6522	0.6547	**0.7225**	Friedman	<0.001	0.405
Partition Ratio	0.8148	0.8141	**0.8626**	Friedman	<0.001	0.409
Anisotropy	0.5483	0.5491	**0.6719**	RM-ANOVA	<0.001	0.608
Trajectory Dir.	0.7076	0.7061	**0.8473**	Friedman	<0.001	0.355
Noise Resilience	0.8486	0.8553	**0.9876**	Friedman	<0.001	0.216
Core Quality	0.7017	0.7034	**0.7855**	Friedman	<0.001	0.480
Overall Quality	0.7328	0.7346	**0.8445**	Friedman	<0.001	0.426

Supplementary Table A3. Comprehensive benchmarking of LAIOR against eleven state-of-the-art methods across 53 scRNA-seq datasets. Performance is evaluated across 22 metrics covering clustering, embedding quality, and latent space continuity. LAIOR demonstrates a dominant advantage in continuity metrics (e.g., 18% improvement in overall quality over siVAE) and noise resilience, while remaining highly competitive in discrete clustering tasks (within 17% of the top-performing scGCC). Methods are grouped by architectural category: traditional clustering, standard variational autoencoders, graph-based methods, deep hierarchical models, and trajectory-specialized methods. Values represent mean scores across all datasets, with the best performance per metric highlighted in bold.

Metric	CLEAR	CellBlast	SCALEX	scVI	scDiffusion	scGNN	scDHMAP	scDeepCluster	scGCC	scTour	siVAE	LAIOR
Clustering and Coupling												
NMI	0.538	0.098	0.123	0.231	0.048	0.457	0.561	0.525	**0.643**	0.191	0.452	0.537
ARI	0.515	0.054	0.080	0.199	0.002	0.430	0.539	0.502	**0.625**	0.157	0.425	0.514
ASW	0.173	0.068	0.087	0.255	0.130	0.284	0.362	0.279	**0.489**	0.181	0.329	0.320
DAV	1.742	1.997	1.926	1.395	1.661	1.188	0.986	1.103	**0.769**	1.787	0.991	1.078
CAL	47.54	24.97	26.14	90.94	63.79	178.9	438.9	193.7	**943.1**	284.2	580.5	765.3
COR	0.811	0.889	0.931	1.631	1.252	3.046	2.151	4.308	4.423	4.178	4.683	**5.715**
Embedding Quality: UMAP												
DC (umap)	0.440	0.356	0.345	0.560	0.682	0.680	0.742	0.715	0.752	**0.890**	0.742	0.889
Q_{local} (umap)	0.467	0.254	0.252	0.527	0.415	0.540	0.634	0.553	**0.666**	0.562	0.611	0.646
Q_{global} (umap)	0.699	0.679	0.677	0.753	0.790	0.765	0.818	0.813	0.825	**0.891**	0.840	**0.891**
Overall (umap)	0.536	0.430	0.425	0.613	0.629	0.662	0.731	0.694	0.748	0.781	0.731	**0.809**
Embedding Quality: t-SNE												
DC (tsne)	0.441	0.472	0.483	0.582	0.619	0.732	0.742	0.721	0.789	0.897	0.824	**0.928**
Q_{local} (tsne)	0.514	0.229	0.229	0.570	0.384	0.621	0.673	0.612	**0.709**	0.574	0.658	0.658
Q_{global} (tsne)	0.695	0.648	0.651	0.760	0.731	0.797	0.822	0.808	0.848	0.893	0.873	**0.911**
Overall (tsne)	0.550	0.450	0.454	0.637	0.578	0.717	0.746	0.713	0.782	0.788	0.785	**0.832**
Continuity Quality: Latent Space												
Manifold Dim.	0.364	0.371	0.372	0.417	0.539	0.603	0.762	0.730	0.776	0.742	0.815	**0.885**
Spectral Decay	0.387	0.391	0.395	0.447	0.544	0.569	0.631	0.711	0.672	0.647	0.677	**0.723**
Partition Ratio	0.120	0.136	0.150	0.348	0.552	0.645	0.710	0.762	0.785	0.803	0.803	**0.863**
Anisotropy	0.109	0.118	0.126	0.251	0.511	0.446	**0.672**	0.663	0.551	0.517	0.566	**0.672**
Trajectory Dir.	0.170	0.177	0.183	0.257	0.355	0.462	0.476	0.614	0.655	0.732	0.712	**0.847**
Noise Resilience	0.046	0.048	0.050	0.081	0.158	0.242	0.432	0.420	0.625	0.610	0.729	**0.988**
Core Quality	0.245	0.254	0.261	0.365	0.537	0.566	0.694	0.716	0.696	0.677	0.715	**0.786**
Overall Quality	0.183	0.190	0.196	0.276	0.406	0.470	0.576	0.626	0.670	0.680	0.717	**0.845**

Supplementary Table A4. Benchmarking of LAIOR against classical disentanglement regularization frameworks. LAIOR's information bottleneck approach is compared to β-VAE, DIP-VAE, β-TC-VAE, and InfoVAE. While traditional disentanglement methods focus on axis-aligned independence, LAIOR significantly outperforms these baselines in manifold continuity and noise resilience (up to 24.7× improvement). Results highlight that classical disentanglement often leads to a collapse in trajectory directionality and spectral properties, whereas LAIOR maintains superior embedding quality on both UMAP and t-SNE projections. All metrics show statistical significance via Friedman tests ($p < 0.001$). Best performance per metric is bolded.

Metric	β-VAE	DIP-VAE	β-TC-VAE	InfoVAE	LAIOR	Test	P-value	ES
Clustering and Coupling								
NMI	0.4201	0.5055	0.5070	0.5352	**0.5365**	Friedman	<0.001	0.393
ARI	0.3921	0.4816	0.4831	0.5127	**0.5137**	RM-ANOVA	<0.001	0.125
ASW	0.1199	0.1602	0.1539	0.1820	**0.3199**	Friedman	<0.001	0.652
DAV	1.9763	1.8086	1.8221	1.6944	**1.0781**	Friedman	<0.001	0.616
CAL	34.1839	41.5041	48.1478	56.1327	**765.2788**	Friedman	<0.001	0.717
COR	0.5753	0.3782	0.6188	1.1027	**5.7153**	Friedman	<0.001	0.754
Embedding Quality: UMAP								
DC (umap)	0.3926	0.3697	0.5138	0.5175	**0.8890**	Friedman	<0.001	0.653
Q_{local} (umap)	0.3484	0.4222	0.4174	0.4670	**0.6458**	Friedman	<0.001	0.698
Q_{global} (umap)	0.6787	0.6846	0.7178	0.7272	**0.8910**	Friedman	<0.001	0.692
Overall (umap)	0.4732	0.4922	0.5497	0.5706	**0.8086**	Friedman	<0.001	0.675
Embedding Quality: t-SNE								
DC (tsne)	0.3864	0.3739	0.5039	0.5135	**0.9277**	Friedman	<0.001	0.683
Q_{local} (tsne)	0.3696	0.4531	0.4425	0.4954	**0.6581**	Friedman	<0.001	0.703
Q_{global} (tsne)	0.6589	0.6762	0.7061	0.7209	**0.9111**	Friedman	<0.001	0.679
Overall (tsne)	0.4716	0.5011	0.5508	0.5766	**0.8323**	Friedman	<0.001	0.688
Continuity Quality: Latent Space								
Manifold Dim.	0.3311	0.3218	0.4035	0.3806	**0.8850**	Friedman	<0.001	0.492
Spectral Decay	0.3743	0.3675	0.3985	0.4061	**0.7225**	Friedman	<0.001	0.688
Partition Ratio	0.0719	0.0504	0.1636	0.2003	**0.8626**	Friedman	<0.001	0.697
Anisotropy	0.0837	0.0683	0.1302	0.1564	**0.6719**	Friedman	<0.001	0.719
Trajectory Dir.	0.1507	0.1387	0.1917	0.1971	**0.8473**	Friedman	<0.001	0.684
Noise Resilience	0.0396	0.0363	0.0533	0.0562	**0.9876**	Friedman	<0.001	0.682
Core Quality	0.2153	0.2020	0.2739	0.2858	**0.7855**	Friedman	<0.001	0.654
Overall Quality	0.1607	0.1499	0.2051	0.2133	**0.8445**	Friedman	<0.001	0.664

Supplementary Table A5. Benchmarking of hyperbolic geometry variants for biological hierarchy modeling. Performance of LAIOR's Lorentz geometry is compared against Poincaré embeddings, Product of Gaussian Manifolds (PGM), Hyperbolic Wrapping (HW), and Lorentz PGM (LPGM). While simple hyperbolic models (Poincaré, PGM) fail to capture the complex, noisy hierarchies of scRNA-seq data, the integrated Lorentz geometry in LAIOR provides substantial improvements in partition ratio (26.7×) and noise resilience (29.9×). The consistent superiority across 20 metrics validates the necessity of Lorentz geometry for stable neural ODE integration in hierarchical biological spaces. Best performance per metric is bolded.

Metric	Poincaré	PGM	HW	LPGM	LAIOR	Test	P-value	ES
Clustering and Coupling								
NMI	0.0448	0.0447	0.0513	0.2735	**0.5365**	RM-ANOVA	<0.001	0.880
ARI	−0.0010	−0.0014	0.0041	0.2383	**0.5137**	RM-ANOVA	<0.001	0.881
ASW	0.0803	0.0785	0.1435	0.0941	**0.3199**	Friedman	<0.001	0.671
DAV	2.1474	2.0776	1.3814	1.9828	**1.0781**	Friedman	<0.001	0.696
CAL	24.2790	25.9613	54.1792	41.0450	**765.2788**	Friedman	<0.001	0.728
COR	0.3521	0.3400	0.5057	0.3959	**5.7153**	Friedman	<0.001	0.569
Embedding Quality: UMAP								
DC (umap)	0.3207	0.3472	0.4821	0.5295	**0.8890**	Friedman	<0.001	0.716
Q_{local} (umap)	0.2477	0.2554	0.3486	0.2856	**0.6458**	Friedman	<0.001	0.632
Q_{global} (umap)	0.6454	0.6563	0.6926	0.6911	**0.8910**	Friedman	<0.001	0.695
Overall (umap)	0.4046	0.4196	0.5078	0.5021	**0.8086**	Friedman	<0.001	0.721
Embedding Quality: t-SNE								
DC (tsne)	0.3088	0.3902	0.4998	0.5323	**0.9277**	Friedman	<0.001	0.745
Q_{local} (tsne)	0.2829	0.2574	0.3725	0.3038	**0.6581**	Friedman	<0.001	0.685
Q_{global} (tsne)	0.6105	0.6241	0.6767	0.6663	**0.9111**	Friedman	<0.001	0.725
Overall (tsne)	0.4007	0.4239	0.5163	0.5008	**0.8323**	Friedman	<0.001	0.725
Continuity Quality: Latent Space								
Manifold Dim.	0.3221	0.3379	0.3828	0.4370	**0.8850**	Friedman	<0.001	0.598
Spectral Decay	0.3589	0.3624	0.4010	0.4126	**0.7225**	Friedman	<0.001	0.754
Partition Ratio	0.0243	0.0322	0.1691	0.2327	**0.8626**	Friedman	<0.001	0.747
Anisotropy	0.0478	0.0560	0.1316	0.1456	**0.6719**	Friedman	<0.001	0.748
Trajectory Dir.	0.1270	0.1326	0.1992	0.2211	**0.8473**	Friedman	<0.001	0.754
Noise Resilience	0.0326	0.0339	0.0580	0.0648	**0.9876**	Friedman	<0.001	0.752
Core Quality	0.1883	0.1971	0.2711	0.3070	**0.7855**	Friedman	<0.001	0.729
Overall Quality	0.1387	0.1451	0.2069	0.2328	**0.8445**	Friedman	<0.001	0.745

Supplementary Table A6. Generalization of the LAIOR model to the scATAC-seq modality across 65 datasets. This table evaluates performance on chromatin accessibility data, which is characterized by higher sparsity and variance compared to RNA-seq. LAIOR is compared against both general (scVI, scTour) and ATAC-specialized (PoissonVI, PeakVI) methods. LAIOR demonstrates consistent superiority, particularly in latent space continuity and noise resilience, where it achieves up to a 95% improvement over the next-best trajectory-optimized method. These results confirm that the integration of Lorentz geometry and neural ODEs provides a robust manifold for inherently noisy epigenetic signals. Bold values indicate the best performance per metric.

Metric	PoissonVI	scVI	PeakVI	scTour	LAIOR	Test	P-value	ES
Clustering and Coupling								
NMI	0.1239	0.1775	0.1299	0.1258	**0.2073**	Friedman	<0.001	0.260
ARI	0.0916	0.1471	0.0977	0.0934	**0.1763**	Friedman	<0.001	0.282
ASW	0.1809	0.1904	0.2006	0.1460	**0.2842**	Friedman	<0.001	0.498
DAV	1.7261	1.6486	1.6079	1.8813	**1.1412**	Friedman	<0.001	0.508
CAL	94.9889	114.3856	175.2359	218.0132	**962.1329**	Friedman	<0.001	0.562
COR	2.0695	2.4598	3.1206	4.0912	**6.3104**	Friedman	<0.001	0.577
Embedding Quality: UMAP								
DC (umap)	0.7253	0.7736	0.7941	0.8458	**0.9357**	Friedman	<0.001	0.392
Q_{local} (umap)	0.4938	0.5261	0.5332	0.5186	**0.6650**	Friedman	<0.001	0.429
Q_{global} (umap)	0.8034	0.8284	0.8372	0.8765	**0.9228**	Friedman	<0.001	0.455
Overall (umap)	0.6742	0.7094	0.7215	0.7470	**0.8412**	Friedman	<0.001	0.471
Embedding Quality: t-SNE								
DC (tsne)	0.7074	0.7498	0.7935	0.8409	**0.9481**	Friedman	<0.001	0.504
Q_{local} (tsne)	0.5087	0.5387	0.5423	0.5259	**0.6681**	Friedman	<0.001	0.359
Q_{global} (tsne)	0.7878	0.8176	0.8247	0.8693	**0.9310**	Friedman	<0.001	0.508
Overall (tsne)	0.6680	0.7020	0.7202	0.7454	**0.8491**	Friedman	<0.001	0.514
Continuity Quality: Latent Space								
Manifold Dim.	0.5213	0.5764	0.6314	0.7025	**0.9034**	Friedman	<0.001	0.573
Spectral Decay	0.4830	0.5129	0.5630	0.6224	**0.7483**	Friedman	<0.001	0.642
Partition Ratio	0.4833	0.5727	0.6368	0.7564	**0.8758**	Friedman	<0.001	0.641
Anisotropy	0.2851	0.3244	0.4019	0.4941	**0.7107**	Friedman	<0.001	0.618
Trajectory Dir.	0.3411	0.3999	0.4914	0.6903	**0.9019**	Friedman	<0.001	0.648
Noise Resilience	0.1298	0.1718	0.3000	0.5040	**0.9820**	Friedman	<0.001	0.592
Core Quality	0.4432	0.4966	0.5583	0.6439	**0.8095**	Friedman	<0.001	0.620
Overall Quality	0.3499	0.4026	0.4866	0.6298	**0.8717**	Friedman	<0.001	0.634

Supplementary Table A7. Sensitivity analysis of Lorentz curvature within the full LAIOR model. Evaluated across 53 scRNA-seq datasets, this table shows how varying the curvature parameter affects model performance. The inclusion of ODE regularization decouples clustering performance from curvature, allowing for high-curvature optimization of continuity metrics (such as manifold dimension and trajectory direction) without degrading cluster separation. High curvature provides significant gains in latent space stability and noise resilience. Bold values indicate the best performance for each metric.

Metric	Lorentz1	Lorentz5	Lorentz10	Test	P-value	ES
Clustering and Coupling						
NMI	**0.5488**	0.5365	0.5245	Friedman	0.0877	0.018
ARI	**0.5268**	0.5137	0.5012	Friedman	0.0613	0.023
ASW	0.2874	0.3199	**0.3470**	Friedman	<0.001	0.377
DAV	1.2509	1.0781	**0.9896**	Friedman	<0.001	0.404
CAL	495.5386	765.2788	**985.2705**	Friedman	<0.001	0.533
COR	4.5762	5.7153	**6.2130**	Friedman	<0.001	0.405
Embedding Quality: UMAP						
DC (umap)	0.8843	**0.8890**	0.8885	Friedman	0.1796	0.009
Q_{local} (umap)	0.6211	0.6458	**0.6614**	Friedman	<0.001	0.294
Q_{global} (umap)	0.8848	0.8910	**0.8938**	Friedman	0.0964	0.017
Overall (umap)	0.7967	0.8086	**0.8146**	Friedman	0.0001	0.101
Embedding Quality: t-SNE						
DC (tsne)	0.9166	**0.9277**	0.9237	Friedman	0.0095	0.047
Q_{local} (tsne)	0.6391	0.6581	**0.6735**	Friedman	<0.001	0.241
Q_{global} (tsne)	0.9017	0.9111	**0.9120**	Friedman	0.0143	0.042
Overall (tsne)	0.8192	0.8323	**0.8364**	Friedman	<0.001	0.141
Continuity Quality: Latent Space						
Manifold Dim.	0.8035	0.8850	**0.8870**	Friedman	<0.001	0.455
Spectral Decay	0.6569	0.7225	**0.7393**	Friedman	<0.001	0.439
Partition Ratio	0.8147	**0.8626**	0.8585	Friedman	<0.001	0.392
Anisotropy	0.5616	0.6719	**0.7087**	Friedman	<0.001	0.525
Trajectory Dir.	0.7068	0.8473	**0.8748**	Friedman	<0.001	0.373
Noise Resilience	0.8833	**0.9876**	0.9749	Friedman	<0.001	0.254
Core Quality	0.7092	0.7855	**0.7984**	Friedman	<0.001	0.465
Overall Quality	0.7433	0.8445	**0.8566**	Friedman	<0.001	0.420

Supplementary Table A8. Impact of Lorentz curvature on the Li-Attn model without ODE regularization. In the absence of neural ODE stabilization, model performance exhibits a much stronger dependency on curvature settings. While high curvature ($c^{-1/2} = 10$) significantly improves trajectory directionality and noise resilience, it also increases computational sensitivity. Comparing these results to the full LAIOR model (Supplementary Table A7) highlights how ODE integration enables curvature decoupling and more stable latent manifold estimation. Bold values indicate the best performance per metric.

Metric	Lorentz1	Lorentz5	Lorentz10	Test	P-value	ES
Clustering and Coupling						
NMI	**0.5546**	0.5511	0.5443	Friedman	0.4791	0.000
ARI	**0.5328**	0.5291	0.5219	Friedman	0.4443	0.000
ASW	0.2188	0.2354	**0.2472**	RM-ANOVA	<0.001	0.046
DAV	1.4584	1.3331	**1.2699**	Friedman	<0.001	0.214
CAL	103.2246	152.9733	**197.4009**	Friedman	<0.001	0.476
COR	2.3387	3.5707	**4.1475**	RM-ANOVA	<0.001	0.583
Embedding Quality: UMAP						
DC (umap)	0.6812	0.7546	**0.8044**	Friedman	<0.001	0.381
Q_{local} (umap)	0.5140	0.5469	**0.5751**	RM-ANOVA	<0.001	0.209
Q_{global} (umap)	0.7904	0.8250	**0.8514**	Friedman	<0.001	0.346
Overall (umap)	0.6618	0.7088	**0.7437**	RM-ANOVA	<0.001	0.321
Embedding Quality: t-SNE						
DC (tsne)	0.6900	0.7636	**0.7984**	Friedman	<0.001	0.297
Q_{local} (tsne)	0.5443	0.5682	**0.5884**	Friedman	<0.001	0.151
Q_{global} (tsne)	0.7930	0.8264	**0.8469**	Friedman	<0.001	0.366
Overall (tsne)	0.6758	0.7194	**0.7446**	Friedman	<0.001	0.294
Continuity Quality: Latent Space						
Manifold Dim.	0.5777	0.6824	**0.7381**	Friedman	<0.001	0.525
Spectral Decay	0.4945	0.5672	**0.6000**	RM-ANOVA	<0.001	0.629
Partition Ratio	0.5482	0.7137	**0.7601**	Friedman	<0.001	0.533
Anisotropy	0.3071	0.4346	**0.5029**	RM-ANOVA	<0.001	0.665
Trajectory Dir.	0.3704	0.5220	**0.5865**	RM-ANOVA	<0.001	0.592
Noise Resilience	0.1731	0.4395	**0.6407**	Friedman	<0.001	0.497
Core Quality	0.4819	0.5995	**0.6503**	RM-ANOVA	<0.001	0.682
Overall Quality	0.3867	0.5442	**0.6292**	RM-ANOVA	<0.001	0.639

Supplementary Table A9. Assessment of likelihood distribution robustness for scRNA-seq analysis. This table evaluates the performance of LAIOR across four different likelihood specifications: Negative Binomial (NB), Zero-Inflated NB (ZINB), Poisson, and Zero-Inflated Poisson (ZIP). Across 53 datasets, the latent representation quality remains largely invariant to the specific distribution choice, with minimal effect sizes observed for continuity and embedding metrics. These results suggest that the architectural refinements of LAIOR (Lorentz mapping and ODE regularization) dominate over the likelihood specification for RNA-seq data. Bold values indicate the best performance per metric.

**Metric**	**NB**	**ZINB**	**Poisson**	**ZIP**	**Test**	**P-value**	**ES**
Clustering and Coupling							
NMI	0.5365	0.5396	0.5526	**0.5544**	RM-ANOVA	0.0011	0.005
ARI	0.5137	0.5171	0.5306	**0.5325**	RM-ANOVA	0.0011	0.005
ASW	0.3199	0.3084	**0.3253**	0.3079	Friedman	0.0011	0.063
DAV	1.0781	1.1203	**1.0730**	1.1310	Friedman	0.0026	0.054
CAL	765.2788	755.5437	**774.4079**	650.3263	Friedman	0.0058	0.046
COR	**5.7153**	5.7135	5.6917	5.6592	Friedman	0.6706	0.000
Embedding Quality: UMAP							
DC (umap)	0.8890	0.8879	0.8996	**0.9029**	Friedman	0.7994	0.000
Q_{local} (umap)	0.6458	0.6386	**0.6535**	0.6369	Friedman	0.0841	0.018
Q_{global} (umap)	0.8910	0.8934	**0.8958**	0.8927	Friedman	0.7994	0.000
Overall (umap)	0.8086	0.8066	**0.8163**	0.8108	Friedman	0.3881	0.000
Embedding Quality: t-SNE							
DC (tsne)	0.9277	0.9247	**0.9292**	0.9170	Friedman	0.1861	0.009
Q_{local} (tsne)	0.6581	**0.6588**	0.6573	0.6492	Friedman	0.7557	0.000
Q_{global} (tsne)	**0.9111**	0.9101	**0.9111**	0.9039	Friedman	0.0474	0.024
Overall (tsne)	0.8323	0.8312	**0.8325**	0.8234	Friedman	0.1190	0.014
Continuity Quality: Latent Space							
Manifold Dim.	0.8850	**0.8911**	0.8811	0.8825	Friedman	0.4058	0.000
Spectral Decay	0.7225	**0.7317**	0.7212	0.7240	Friedman	0.7666	0.000
Partition Ratio	0.8626	**0.8709**	0.8601	0.8655	Friedman	0.2769	0.004
Anisotropy	**0.6719**	0.6713	0.6715	0.6591	Friedman	0.6654	0.000
Trajectory Dir.	0.8473	**0.8814**	0.8415	0.8607	Friedman	0.2296	0.006
Noise Resilience	**0.9876**	0.9819	0.9838	0.9793	Friedman	0.2177	0.007
Core Quality	0.7855	**0.7913**	0.7835	0.7828	Friedman	0.6298	0.000
Overall Quality	0.8445	**0.8565**	0.8410	0.8455	Friedman	0.5305	0.000

Supplementary Table A10. Assessment of modality-specific likelihood effects for scATAC-seq analysis. Unlike scRNA-seq, chromatin accessibility data shows a high sensitivity to likelihood distribution. Zero-Inflated Poisson (ZIP) significantly improves discrete clustering performance (NMI/ARI) by sharpening boundaries in sparse data. However, the standard Negative Binomial (NB) distribution is superior for maintaining latent manifold continuity and global embedding quality. These findings suggest that while zero-inflation models can aid cluster separation in ATAC data, they may distort continuous trajectory properties. Bold values indicate the best performance per metric.

Metric	NB	ZINB	Poisson	ZIP	Test	P-value	ES
Clustering and Coupling							
NMI	0.2073	0.2897	0.2128	**0.3072**	Friedman	<0.001	0.421
ARI	0.1763	0.2618	0.1819	**0.2800**	Friedman	<0.001	0.421
ASW	0.2842	0.2908	0.2774	**0.2950**	Friedman	0.3282	0.002
DAV	1.1412	**1.0961**	1.2126	1.1132	Friedman	0.4954	0.000
CAL	**962.1329**	718.3740	904.8563	760.8987	Friedman	<0.001	0.134
COR	**6.3104**	6.0081	5.9839	6.0744	Friedman	0.2761	0.003
Embedding Quality: UMAP							
DC (umap)	**0.9357**	0.9306	0.9045	0.9196	Friedman	0.0005	0.058
Q_{local} (umap)	**0.6650**	0.6576	0.6499	0.6558	Friedman	0.4150	0.000
Q_{global} (umap)	**0.9228**	0.9167	0.9123	0.9134	Friedman	0.0021	0.046
Overall (umap)	**0.8412**	0.8349	0.8222	0.8296	Friedman	0.0081	0.034
Embedding Quality: t-SNE							
DC (tsne)	**0.9481**	0.9331	0.9180	0.9237	Friedman	<0.001	0.103
Q_{local} (tsne)	**0.6681**	0.6570	0.6450	0.6524	Friedman	0.0120	0.031
Q_{global} (tsne)	**0.9310**	0.9179	0.9176	0.9157	Friedman	<0.001	0.136
Overall (tsne)	**0.8491**	0.8360	0.8269	0.8306	Friedman	<0.001	0.092
Continuity Quality: Latent Space							
Manifold Dim.	**0.9034**	0.8992	0.8777	0.8967	Friedman	0.0666	0.016
Spectral Decay	**0.7483**	0.7424	0.7316	0.7437	Friedman	0.0392	0.021
Partition Ratio	**0.8758**	0.8735	0.8498	0.8746	Friedman	0.1001	0.013
Anisotropy	**0.7107**	0.6879	0.6837	0.6942	Friedman	0.0033	0.042
Trajectory Dir.	**0.9019**	0.8891	0.8754	0.8939	Friedman	0.1305	0.010
Noise Resilience	0.9820	**0.9950**	0.9373	0.9809	Friedman	0.0419	0.020
Core Quality	**0.8095**	0.8007	0.7857	0.8023	Friedman	0.0263	0.024
Overall Quality	**0.8717**	0.8661	0.8429	0.8655	Friedman	0.0946	0.013

Supplementary Table A11. Latent factor decomposition across three biological systems reveals interpretable modules aligned to cell cycle, lineage identity, and functional programs. LAIOR's bottleneck pathway captures coordinated biological factors rather than statistically independent components. Each dataset exhibits distinct modular organization: human bone marrow (5 major modules spanning proliferation, B-cell maturation, granulocytes, myeloid cells, and antigen presentation), mouse pancreatic endocrinogenesis (5 modules capturing progenitor specification, islet lineages, beta cell function, exocrine tissue, and stress response), and *Dapp1* knockout hematopoietic cells (5 modules revealing lineage-biased progenitor states: MEP, GMP, CLP, cell cycle, and HSC maintenance).

Dataset	Group	Latent Factors	Biological Identity	Key Genes
**Human Bone Marrow (10 latent factors)**				
	1	**L3, L8**	Cell Cycle & Proliferation (S-phase and G2/M)	*TYMS, TK1, TUBB*
	**2**	**L1, L2**	B-Cell Lineage (Precursors to Plasma cells)	*IGKC* (Plasma), *VPREB3* (Pre-B)
	**3**	**L0, L9**	Neutrophils & Inflammation (Granulocyte population)	*MPO, CXCL8*
	**4**	**L5, L7**	Myeloid / Macrophages (Lipid metabolism)	*APOC1, CSF1*
	**5**	**L4, L6**	Antigen Presentation & Stroma (Dendritic cells and stromal support)	*IRF8* (DC), *WNT2B* (Stroma)
**Mouse Pancreatic Endocrinogenesis (10 latent factors)**				
	1	**L1, L3**	Endocrine Progenitors (Islet precursor commitment)	*Gast, Sox4, Tgm2*
	**2**	**L6, L8**	Alpha & Delta Lineages (Non-beta islet cells)	*Irx1* (Alpha), *Sst* (Delta)
	**3**	**L0, L4, L9, L7**	Functional Beta Cell Core (Pan-endocrine identity, hormone processing, proteostasis)	*Nnat, Isl1, Pcsk2, Ctxn2, Ubqln2*
	**4**	**L5**	Exocrine Tissue (Acinar cells)	*Serpina1a, Fabp3*
	**5**	**L2**	Cellular Stress (Dissociation artifact)	*Fos*
***Dapp1* Knockout Mouse LSK Cells (10 latent factors)**				
	1	**L1, L3**	MEP Biased (Erythroid/Megakaryocyte)	*Gata1* (Master Regulator), *Car2* (Erythroid), *Nedd4*
	**2**	**L2, L9**	GMP Biased (Granulocyte/Macrophage)	*Mpo* (Myeloid Primer), *Lgsf6*, *Mt1*
	**3**	**L5, L7**	Cell Cycle (G2/M Phase) (Proliferative fraction)	*Birc5* (Survivin), *Ccnb2* (Cyclin), *Tuba1b*
	**4**	**L4, L8**	CLP Biased (Lymphoid potential)	*Ighm* (Ig Heavy Chain), *Gm11808*
	**5**	**L0, L6**	HSC Maintenance & Baseline (Housekeeping state)	*Marcksl1* (Cytoskeleton), *Ptma*, *Rps27a*

Supplementary Table A12. Single-cell datasets used for trajectory inference validation span diverse hematopoietic and developmental contexts.

GEO Accession	Biological System & Modality	Main Context	Link to Development / Continuous States
**GSE120446**	Adult human bone marrow; scRNA-seq + mass/flow cytometry	Normal hematopoiesis	Strong: captures continuous hematopoietic differentiation from HSCs through all major mature lineages
**GSE123902**	Human lung adenocarcinoma; scRNA-seq (10x) from primary tumors, metastases, and normal tissue	Solid tumor (LUAD)	Strong but disease-focused: tumor progression continuum (normal → malignant, primary → metastasis, EMT, stem-like states)
**GSE130148**	Adult normal human lung; scRNA-seq	Non-malignant lung atlas	Moderate: steady-state differentiation/activation gradients (airway–alveolar epithelial, immune activation)
**GSE247719**	Mouse multi-tissue single-nucleus RNA-seq across ages (PanSci)	Organismal aging	Very strong: models continuous aging trajectories within and across cell types, revealing age-emergent sub-populations
**GSE283205**	Pediatric hepatoblastoma; snRNA-seq from FFPE tumors	Pediatric liver cancer	Strong and explicitly continuous: tumor cells on spectrum of hepatic differentiation programs; Wnt–MDK signaling drives immune-evasive microenvironment

Supplementary Table A13. Detailed computational benchmarking across 17 scRNA-seq cancer datasets. Training performed with 3,000 highly variable genes, batch sizes scaled to cell count, and 400 maximum epochs with early stopping. Datasets ordered by cell count (ascending). Methods ordered by computational cost (ascending). Time in seconds; peak memory in GB. Hardware: NVIDIA RTX 5090 24 GB GPU. Architectural variants: scVI (baseline scVI-tools), iVAE (information bottleneck regularization), LiVAE (Lorentz geometric constraints), Li-Attn (attention mechanism added), Li-Attn+ODE (full LAIOR with neural ODE dynamics).

**Datasets 1–9 (7,431–27,796 cells)**					
**Dataset**	**Cells**	**Method**	**Batch**	**Time (s)**	**Mem (GB)**
GSE117988 Tumor	7,431	scVI	192	55.4	0.321
		iVAE	192	79.2	0.339
		LiVAE	192	91.7	0.354
		Li-Attn	192	119.3	0.522
		Li-Attn+ODE	192	848.4	0.646
GSE155109 Breast EC	8,433	scVI	218	57.3	0.366
		iVAE	218	66.6	0.385
		LiVAE	218	68.1	0.399
		Li-Attn	218	113.2	0.575
		Li-Attn+ODE	218	969.5	0.697
GSE117988 PBMC	12,874	scVI	333	57.7	0.093
		iVAE	333	89.1	0.120
		LiVAE	333	111.8	0.136
		Li-Attn	333	149.0	0.348
		Li-Attn+ODE	333	1367.2	0.481
GSE149655 Cervical	13,060	scVI	338	58.5	0.346
		iVAE	338	103.9	0.374
		LiVAE	338	108.2	0.389
		Li-Attn	338	165.3	0.603
		Li-Attn+ODE	338	1432.9	0.734
GSE283205 Hepato	16,506	scVI	427	59.0	0.358
		iVAE	427	110.4	0.392
		LiVAE	427	115.1	0.406
		Li-Attn	427	159.4	0.648
		Li-Attn+ODE	427	1794.7	0.787
GSE225857 Liver Meta	22,260	scVI	577	67.6	0.383
		iVAE	577	147.2	0.428
		LiVAE	577	152.7	0.433
		Li-Attn	577	240.1	0.722
		Li-Attn+ODE	577	2426.1	0.873
GSE168181 Breast	23,556	scVI	610	64.7	0.388
		iVAE	610	155.2	0.436
		LiVAE	610	160.8	0.442
		Li-Attn	610	247.9	0.740
		Li-Attn+ODE	610	2562.4	0.893
GSE123813 SCC	26,016	scVI	674	76.5	0.401
		iVAE	674	164.1	0.453
		LiVAE	674	168.4	0.457
		Li-Attn	674	273.0	0.774
		Li-Attn+ODE	674	2826.8	0.932
GSE124310 Myeloma	27,796	scVI	720	69.2	0.409
		iVAE	720	172.3	0.465
		LiVAE	720	173.3	0.470
		Li-Attn	720	279.7	0.803
		Li-Attn+ODE	720	3000.6	0.963

**Datasets 10–17 (32,349–62,035 cells)**					
**Dataset**	**Cells**	**Method**	**Batch**	**Time (s)**	**Mem (GB)**
GSE228499 Breast	32,349	scVI	838	75.6	0.428
		iVAE	838	176.0	0.495
		LiVAE	838	178.6	0.500
		Li-Attn	838	268.7	0.866
		Li-Attn+ODE	838	3426.5	1.036
GSE138709 Liver	33,991	scVI	881	91.2	0.432
		iVAE	881	202.6	0.500
		LiVAE	881	202.4	0.504
		Li-Attn	881	354.3	0.889
		Li-Attn+ODE	881	3682.6	1.062
GSE132509 Leukemia	39,375	scVI	1020	73.8	0.250
		iVAE	1020	262.8	0.329
		LiVAE	1020	252.8	0.342
		Li-Attn	1020	383.1	0.763
		Li-Attn+ODE	1020	4291.9	0.945
GSE123902 Lung Adre	42,847	scVI	1110	88.0	0.508
		iVAE	1110	338.0	0.592
		LiVAE	1110	346.0	0.598
		Li-Attn	1110	445.8	1.054
		Li-Attn+ODE	1110	4659.2	1.242
GSE189357 Lung Adre	45,938	scVI	1190	85.5	0.484
		iVAE	1190	324.0	0.574
		LiVAE	1190	334.4	0.578
		Li-Attn	1190	532.3	1.059
		Li-Attn+ODE	1190	4988.5	1.255
GSE123813 BCC	53,030	scVI	1374	97.8	0.512
		iVAE	1374	373.0	0.615
		LiVAE	1374	389.5	0.621
		Li-Attn	1374	599.8	1.158
		Li-Attn+ODE	1374	5844.0	1.367
GSE163558 Stomach	54,687	scVI	1417	96.7	0.520
		iVAE	1417	383.3	0.625
		LiVAE	1417	378.5	0.631
		Li-Attn	1417	635.7	1.181
		Li-Attn+ODE	1417	6030.5	1.392
GSE183904 Gastric	62,035	scVI	1608	95.5	0.592
		iVAE	1608	384.3	0.712
		LiVAE	1608	241.9	0.717
		Li-Attn	1608	613.6	1.325
		Li-Attn+ODE	1608	6851.8	1.553
