# Supplementary material for: LAIOR: a hyperbolic neural ODE variational framework for interpretable single-cell manifold learning and trajectory inference
Source: Front Genet. 2026 Jun 8;17:1838613. doi: 10.3389/fgene.2026.1838613 (PMC13283489; doi:10.3389/fgene.2026.1838613)
Supplement: Supplementary file 2 [file Supplementaryfile2.pdf]

Supplementary Table 1: Summary of scRNA-seq Datasets Used in This Study

Comprehensive summary of 48 single-cell RNA-seq (scRNA-seq) studies representing 53 distinct samples analyzed in this meta-analysis. The table provides study identifiers (GEO Series Accession numbers), comprehensive experimental details including sequencing platform, organism source, tissue/cell type origin, and key methodological and biological findings. Studies encompass diverse biological contexts spanning cancer immunology (22 studies), developmental biology and normal tissue characterization (25 studies), and cell differentiation processes (1 algorithmic study). Tissue diversity includes bone marrow, lung, brain, immune cells, epithelial tissues, and embryonic cell systems. Platform diversity reflects contemporary high-throughput scRNA-seq technologies. Sample-level identifiers and representation details are presented in Supplementary Table 4.

GSE #	GSE Accession	Study Title	Species	Platform	Primary Tissue	Key Findings
1	GSE120221	Human Bone Marrow Assessment by Single Cell RNA Sequencing, Mass Cytometry and Flow Cytometry	Homo sapiens	HiSeq 3000	Bone marrow	20 donors; 880 genes/cell; multi-platform validation
2	GSE115571	Single-cell transcriptomics reveals distinct microglia signatures under inflammation	Mus musculus	NextSeq 500	Microglia	LPS vs. control; inflammatory signatures
3	GSE130148	Single cell RNA sequencing of fresh resected human lung tissue	Homo sapiens	HiSeq 4000	Lung parenchyma	4 patients; Drop-seq; airway + distal tissues
4	GSE142653	Developmental lineage trajectories during human pituitary development	Homo sapiens	HiSeq 2500	Fetal pituitary	4,113 cells; lineage mapping; developmental trajectories
5	GSE145861	Single-cell RNA-sequencing of adult mouse prostates	Mus musculus	NextSeq 500	Prostate	10X Genomics; normal tissue atlas
6	GSE145865	Single-cell RNA-sequencing of adult mouse urethra	Mus musculus	NextSeq 500	Urethra	10X Genomics; normal tissue characterization
7	GSE165784	Microglia heterogeneity in diabetic retinopathy and vitreoretinopathy	Mus musculus	HiSeq X Ten	Fibrous membrane (retina)	1 PVR + 3 PDR patients; scFTD-seq; microglia profiling
8	GSE189070	Astrocyte heterogeneity following spinal cord injury	Mus musculus	NovaSeq 6000	Spinal cord	7 timepoints post-injury; injury response trajectories
9	GSE213740	Macrophage involvement in aortic dissection	Homo sapiens	NextSeq 500	Aortic wall	6 disease + 3 control; macrophage function

GSE #	GSE Accession	Study Title	Species	Platform	Primary Tissue	Key Findings
		pathogenesis				
10	GSE247719	Cell population dynamics across mammalian aging (Pan-tissue)	Mus musculus	NovaSeq 6000	Multi-tissue (20+)	Massive aging atlas; multiple genotypes; 3-23 months
11	GSE275119	SFRP2+ fibroblast progenitors in tooth development	Mus musculus	NovaSeq 6000	Incisor pulp	15 mice; developmental stages; fibroblast dynamics
12	GSE132188	Pancreatic endocrinogenesis developmental roadmap	Mus musculus	HiSeq 4000	Pancreas	Ngn3-Venus reporter; E12.5-E15.5 timepoints; 36,351 cells
13	GSE253355	Cellular biogeography of human bone marrow niches	Homo sapiens	NovaSeq 6000	Bone marrow	Fresh femoral head; mesenchymal + HSPC populations
14	GSE167597	Cholinergic neuron diversity in adult spinal cord	Mus musculus	HiSeq 2500	Spinal cord	Cholinergic nuclei; FACS sorting; neuron type diversity
15	GSE144024	Human megakaryocyte development	Homo sapiens	HiSeq X Ten	Embryonic HSCs	50,000+ cells; megakaryocyte heterogeneity
16	GSE192857	hESC neuronal differentiation time series	Homo sapiens	NovaSeq 6000	hESC-neurons	Day 0-20; 19 samples; differentiation trajectory
17	GSE255019	Age-specific platelet differentiation from HSCs	Mus musculus	NovaSeq 6000	Bone marrow	Age-dependent differentiation; clonal analysis
18	GSE226131	HSCs from young and aged mice	Mus musculus	MiSeq	Bone marrow	DRAG mice; FACS-sorted; aging effects
19	GSE165844	External signals regulate HSC transcriptional states	Mus musculus	NextSeq 500	Bone marrow	Multiple stimuli; dmPGE2, pIC, GCSF; CITE-Seq
20	GSE141259	Lung injury response to bleomycin	Mus musculus	HiSeq 4000	Lung epithelium	Longitudinal timecourse; injury trajectory
21	GSE95753	Neurogenesis in dentate gyrus (adult brain)	Mus musculus	HiSeq 2500	Dentate gyrus	Postnatal days 12-35; temporal dynamics
22	GSE226824	HSPC response to in vivo IFNα treatment	Mus musculus	NovaSeq 6000	Bone marrow	0-72h timecourse; cell hashing; inflammatory response
23	GSE148215	Human definitive hematopoietic stem/progenitor cell generation	Homo sapiens	HiSeq X Ten	hESC-derived HSCs	H1 line; CD43+ differentiation; CD34+CD38- mobilized

GSE #	GSE Accession	Study Title	Species	Platform	Primary Tissue	Key Findings
24	GSE120505	Aging in peripheral blood	Mus musculus	HiSeq 4000	Blood	Young vs. old mice; aging signatures
25	GSE117988	Merkel cell carcinoma immunotherapy resistance	Homo sapiens	HiSeq 2500	Tumor + blood	Serial timepoints; immunotherapy response
26	GSE124310	Immune microenvironment in multiple myeloma progression	Homo sapiens	HiSeq 2500	Bone marrow	40,800 immune cells; 22 MM + 9 healthy; progression stages
27	GSE138709	Intrahepatic cholangiocarcinoma transcriptomic landscape	Homo sapiens	HiSeq X Ten	ICC + adjacent	31,302 cells; 31 cell subtypes; heterogeneity profiling
28	GSE149655	KRAS-mutant lung adenocarcinoma cell states	Homo sapiens	NovaSeq 6000	Lung tumor + normal	2 LUAD patients; tumor/normal comparison
29	GSE163558	Gastric cancer transcriptional heterogeneity and metastasis	Homo sapiens	NovaSeq 6000	Stomach + metastases	42,968 cells; 6 patients; organ-specific metastases
30	GSE168181	Breast cancer lymph node metastasis model	Mus musculus	NextSeq 500	LN + tumor	Spontaneous metastasis; TME dynamics
31	GSE189357	Lung adenocarcinoma progression atlas (AIS→IAC)	Homo sapiens	NovaSeq 6000	Lung adenocarcinoma	9 samples; 3 each AIS/MIA/IAC; spatial transcriptomics
32	GSE225857	CRC liver metastasis cellular heterogeneity	Homo sapiens	NovaSeq 6000	CRC + liver	6 patients; primary/CN/LM/LN/PB; spatial transcriptomics
33	GSE228499	Primary breast cancer scRNA-seq	Homo sapiens	HiSeq 2500	Breast cancer	10X Chromium v2; standard protocol
34	GSE283205	Hepatoblastoma differentiation and immune evasion	Homo sapiens	NovaSeq 6000	Hepatoblastoma (FFPE)	5 pediatric samples; snRNA-seq; Wnt/MDK signaling
35	GSE148218	ETV6-RUNX1 pediatric B-ALL leukemic states	Homo sapiens	HiSeq 3000	BM + cell line	6 patient samples; drug response screening
36	GSE262288	CDK4/6 inhibitor response in HR+/HER2- breast cancer	Homo sapiens	NovaSeq 6000	Metastases (multi-site)	Responders vs. progressors; validated cohorts
37	GSE155109	Lipid-processing endothelial cells in breast cancer	Homo sapiens	HiSeq 4000	Breast stroma + EC	9 patients; EC-enriched + stromal populations

GSE #	GSE Accession	Study Title	Species	Platform	Primary Tissue	Key Findings
38	GSE123813	PD-1 blockade tumor-specific T cell clonal replacement	Homo sapiens	HiSeq 4000	BCC tumor	Immunotherapy response; TCR tracking
39	GSE143423	Immune phenotypes in metastatic brain tumors	Homo sapiens	HiSeq X Ten	Brain metastasis	3 NSCLC + 1 TNBC patients; TME characterization
40	GSE123902	Lung adenocarcinoma transcriptional landscape	Homo sapiens	HiSeq 2500	LUAD ± metastases	17 donors; primary/metastatic comparison
41	GSE225600	Breast cancer primary tumor + LN metastasis	Homo sapiens	NovaSeq 6000	Breast + LN	4 pairs; multimodal (scRNA + spatial)
42	GSE235787	B-ALL drug response prediction (network pharmacology)	Homo sapiens	NovaSeq 6000	Blood	L-asp sensitivity/resistance; PDX models
43	GSE222002	Bystander T cells in tumor immunotherapy	Mus musculus	NovaSeq 6000	Tumor/spleen	LCMV/MC38 model; NDV immunotherapy; TCR-seq
44	GSE98638	T cell infiltration in hepatocellular carcinoma	Homo sapiens	HiSeq 2500	Liver + blood	HCC TILs; immune profiling
45	GSE222369	Nicotinamide-enhanced NK cell function	Homo sapiens	NovaSeq 6000	Blood (NK cells)	Clinical trial; NAM expansion; therapeutic response
46	GSE183904	Gastric cancer lineage states and TME	Homo sapiens	NovaSeq 6000	Stomach (GC)	31 samples; >200,000 cells; 34 lineage states
47	GSE132509	Childhood leukemia developmental heterogeneity	Homo sapiens	HiSeq 4000	BM (ALL)	Multiple ALL subtypes + controls; 21,370 cells
48	S-SUBS8	Cell fate probabilities in Palantir (Human Cell Atlas)	Homo sapiens	Human Cell Atlas	CD34+ HSCs	Algorithm development; continuous differentiation

Supplementary Table 2: Multi-sample GSE Studies and Sample-level Stratification Details

Detailed breakdown of scRNA-seq studies with multiple data representations (53 total samples from 48 GSE projects). This table identifies projects where biological/tissue/disease stratification resulted in multiple files or datasets, enabling sample-level differentiation and cross-study comparison. Five studies contain multiple representations: GSE117988 stratified by tissue source (tumor vs. peripheral blood), GSE123813 stratified by cancer type (BCC vs. SCC), GSE143423 stratified by tumor origin (NSCLC vs. TNBC), GSE155109 stratified by cell population (endothelial vs. stromal), and GSE247719 stratified by tissue type from multi-tissue atlas. These stratifications reflect biological heterogeneity and enable focused analysis of specific tissue compartments or disease phenotypes.

GSE #	GSE Accession	Study Title	N Files	Stratification Basis	Sample Designation	Biological Context
1	GSE117988	Merkel cell carcinoma immunotherapy resistance	2	**Tissue source**	PBMC vs. Tumor	Serial timepoint comparison; PBMC vs. tumor biopsies
2	GSE123813	PD-1 blockade tumor-specific T cells	2	**Cancer type**	BCC vs. SCC	Basal cell carcinoma vs. squamous cell carcinoma histology
3	GSE143423	Metastatic brain tumor immune phenotypes	2	**Tumor origin**	NSCLC (LBM) vs. TNBC	Primary tumor type determines metastatic immune context
4	GSE155109	Endothelial cells in breast cancer	2	**Cell population**	EC-enriched vs. Stromal	Endothelial cells vs. stromal/other cell populations
5	GSE247719	Pan-tissue aging atlas	2	**Tissue type**	Muscle vs. T cells	Multi-tissue atlas; tissue-specific sampling

Supplementary Table 3: Summary of scATAC-seq Datasets Used in This Study

Comprehensive summary of 18 single-cell ATAC-seq (scATAC-seq) studies encompassing 65 samples analyzed in this meta-analysis. The table provides study identifiers (GEO Series Accession numbers), experimental details including sequencing platform, sample organism, tissue source, and key methodological findings. Studies span multiple tissue types and disease contexts, including cancer microenvironment profiling, developmental processes, and tissue regeneration models. Sample identifiers are presented separately in Supplementary Table 2 for detailed reference.

GSE #	GSE Accession	Study Title	Species	Platform	N Samples	Source Tissue	Key Findings
1	GSE274934	Single-cell multi-omics reveals tumor microenvironment factors underlying poor immunotherapy responses in ALK-positive lung cancer	Homo sapiens	HiSeq X Ten	4	Lung	ALK+/WT LUAD; distinct immune landscapes; multi-omics profiling
2	GSE211087	Integrated multiomics profiling identifies the differentiation program of regulatory T cells	Homo sapiens	NovaSeq 6000	3	PBMC & TIL	NSCLC eTreg cells; regulatory T cell differentiation

GSE #	GSE Accession	Study Title	Species	Platform	N Samples	Source Tissue	Key Findings
		in human tumors [scATAC-seq]					
3	GSE168026	Enhancer reactivation mediates adaptive resistance to FGFR inhibitors in triple-negative breast cancer [scATAC-seq]	Homo sapiens	NovaSeq 6000	1	Breast cancer	TNBC PDX; FGFR inhibitor response; enhancer dynamics
4	GSE200813	Single-cell chromatin accessible state of paired primary and liver metastasis colorectal cancer cells from PDX models	Homo sapiens	NovaSeq 6000	4	Colon & Liver	CRC metastasis; primary/metastatic comparison; PDX-derived
5	GSE241745	Single-cell transcription and chromatin accessibility profiles of IDH mutant gliomas	Homo sapiens	NextSeq 500	6	Brain	IDH glioma; primary + organoid; integrated scRNA + scATAC
6	GSE292194	Patterns of intra- and inter-tumor phenotypic heterogeneity in lethal prostate cancer [scATAC-seq]	Homo sapiens	NovaSeq X Plus	3	Prostate tumor	Lethal PCa; intra/inter-tumor heterogeneity; 3 patients
7	GSE178988	Transcriptional programs dictating Schwann cell transformation in MPNST [scATAC-seq]	Homo sapiens, Mus musculus	NovaSeq 6000	2	Peripheral nerve	MPNST; neurofibromatosis; human + mouse models
8	GSE226108	A multi-omics atlas of the human retina at	Homo sapiens	NovaSeq 6000	6	Retina	Multi-omic; 25 donors; peripheral/foveal/macular regions

GSE #	GSE Accession	Study Title	Species	Platform	N Samples	Source Tissue	Key Findings
		single-cell resolution					
9	GSE192947	A regulatory network of Sox and Six transcription factors in hearing regeneration in adult zebrafish	Danio rerio	NextSeq 550	5	Inner ear	Hair cell regeneration; wild-type + DTR ablation; temporal dynamics
10	GSE198730	Transcriptomics, Regulatory Syntax, and Enhancer Identification in Mesoderm-Induced ESCs	Mus musculus	HiSeq 2500	2	ESC-derived tissues	Mouse ESC differentiation; aPSM + naive ESC; chromatin accessibility + Hi-C
11	GSE220026	Single-cell ATAC-seq analysis of hESCs undergoing neuronal differentiation with paracetamol exposure [scATAC-seq]	Homo sapiens	HiSeq 2000	3	hESC-derived neurons	Neuronal differentiation; Day 0-20 timeseries; drug exposure effects
12	GSE201213	Single cell ATAC-seq of mouse hair follicle morphogenesis	Mus musculus	NovaSeq 6000	3	Skin (hair follicle)	23,716 nuclei; developmental stages (E13.5, E16.5, P0)
13	GSE284492	Decoding Gene Networks Governing Hypothalamic and Prethalamic Neuron Development	Mus musculus	NovaSeq 6000	4	Hypothalamus & Prethalamus	Brain development; cortical interneurons; integrated scRNA + scATAC
14	GSE199556	Longitudinal single cell transcriptional and epigenetic mapping of CD8 T cell fates [scATAC-Seq]	Mus musculus	NovaSeq 6000	6	Spleen (P14 CD8+ T cells)	LCMV infection; effector/memory/exhausted states; Days 2-200 post-infection

GSE #	GSE Accession	Study Title	Species	Platform	N Samples	Source Tissue	Key Findings
15	GSE205549	Tissue-resident memory CD8+ T cells: transcriptional and epigenetic heterogeneity [scATAC-seq]	Mus musculus	NovaSeq 6000	1	Small intestine IEL	Tissue-resident memory T cells; heterogeneity analysis
16	GSE243004	Metabolic adaptation pilots differentiation of human hematopoietic cells (scATAC-Seq)	Homo sapiens	NovaSeq 6000	4	Umbilical cord blood	CD34+ HSCs; metabolic inhibitor treatments; 24h culture
17	GSE232002	Inflammatory Cytokines Affect Innate Lymphoid Cell Fate After Allogeneic Stem Cell Transplantation [scATAC-Seq]	Homo sapiens	NextSeq 2000	2	PBMC	Allogeneic HSCT; pre/post-treatment; graft-versus-host disease
18	GSE294221	Chronic Cigarette Smoking on Lung Epithelial Responses to Influenza and Hyperoxia [scATAC-Seq]	Mus musculus	NovaSeq 6000	6	Lung	AKR/J mice; smoke + bleomycin; multiome-seq (scRNA + scATAC)

Supplementary Table 4: Sample-level Identifiers and GSE-GSM Mapping for scATAC-seq Datasets

Detailed mapping of individual sample identifiers (GEO Sample Accession numbers, GSM) to their parent studies (GEO Series Accession numbers, GSE) for all 65 scATAC-seq samples. This reference table enables traceability and identification of specific samples within each study. GSM identifiers are sortable by GSE accession for cross-referencing with raw data repositories (NCBI GEO, ENA). Total of 63 individually mapped GSM samples plus 2 study-level identifiers from GSE198730.

GSE #	GSE Accession	Study	N	GSM Sample Identifiers
1	GSE274934	ALK+ lung cancer multi-omics	4	GSM8462143, GSM8462144, GSM8462145, GSM8462147

GSE #	GSE Accession	Study	N	GSM Sample Identifiers
2	GSE211087	Regulatory T cell differentiation	3	GSM6449878, GSM6449880, GSM6449881
3	GSE168026	TNBC FGFR inhibitor response	1	GSM5124061
4	GSE200813	CRC metastasis primary/LM	4	GSM6044073, GSM6044075, GSM6044076, GSM6044077
5	GSE241745	IDH glioma primary + organoid	6	GSM7734290, GSM7734291, GSM7734292, GSM7734712, GSM7734715, GSM7734716
6	GSE292194	Lethal prostate cancer	3	GSM8852347, GSM8852348, GSM8852349
7	GSE178988	MPNST human + mouse	2	GSM5402770, GSM5402771
8	GSE226108	Retina multi-omic atlas	6	GSM7884540, GSM7884541, GSM7884545, GSM7884549, GSM7884550, GSM7884543
9	GSE192947	Zebrafish hair cell regeneration	5	GSM5769453, GSM5769456, GSM5769461, GSM5769462, GSM5769463
10	GSE198730	Mouse ESC differentiation	2	GSE198730_aPSM_scATAC_rep1, GSE198730_naive_ESC_scATAC_rep1
11	GSE220026	hESC neuronal differentiation	3	GSM5766892, GSM6783751, GSM6783752
12	GSE201213	Mouse hair follicle development	3	GSM6052781, GSM6052782, GSM6052783
13	GSE284492	Mouse brain development	4	GSM8517035, GSM8685152, GSM8685155, GSM8685177
14	GSE199556	CD8 T cell differentiation	6	GSM5975165, GSM5975166, GSM7062446, GSM7062447, GSM7062449, GSM7062450
15	GSE205549	Small intestine Trm cells	1	GSM6214535
16	GSE243004	Hematopoietic metabolic adaptation	4	GSM7777060, GSM7777061, GSM7777062, GSM7777063
17	GSE232002	Allogeneic HSCT ILC differentiation	2	GSM7308367, GSM7308369
18	GSE294221	Cigarette smoke lung response	6	GSM8546124, GSM8546125, GSM8900548, GSM8900549, GSM8900550, GSM8900551

Summary Statistics

scRNA-seq

- **Total studies:** 48 scRNA-seq projects
- **Total samples/files:** 53 (48 projects; 5 projects have two representations; see Supplementary Table 2)

- **Organisms (study-level):** 31 Homo sapiens (64.6%), 16 Mus musculus (33.3%), 1 external non-GEO resource (2.1%; Human Cell Atlas / S-SUBS8)
- **Multi-representation projects (n = 5; each contributes 2 files):** GSE117988 (PBMC vs tumor), GSE123813 (BCC vs SCC), GSE143423 (NSCLC vs TNBC), GSE155109 (EC-enriched vs stromal), GSE247719 (muscle vs T cells)
- **Study themes (not mutually exclusive; curated):** Cancer/Oncology 22 studies (26 samples); Developmental biology 16 (19); Normal tissue/homeostasis 9 (7); Immunology 1 (1); Algorithm/method development 1 (1); Cross-category aging atlas 1 (2)
- **Platforms (high-level):** Predominantly Illumina sequencers (NovaSeq/HiSeq/NextSeq), with a small number of specialized protocols (e.g., Drop-seq, scFTD-seq) and one external curated dataset

scATAC-seq

- **Total studies:** 18 scATAC-seq projects
- **Total samples:** 65 (63 GSM + 2 study-level identifiers from GSE198730; see Supplementary Table 4)
- **Organisms (study-level):** 13 Homo sapiens, 4 Mus musculus, 1 Danio rerio
- **Cross-species studies:** 1 (GSE178988; human + mouse)
- **Study categories (mutually exclusive; curated):** Oncology 9, Developmental biology 6, Immunology 3
- **Platforms (study-level; curated):** NovaSeq 6000 (11), NovaSeq X Plus (1), HiSeq variants (3), NextSeq variants (3)

Combined (scRNA-seq + scATAC-seq)

- **Total studies:** 66
- **Total samples:** 118 (53 scRNA-seq + 65 scATAC-seq)
- **Organisms:** 44 Homo sapiens (66.7%), 20 Mus musculus (30.3%), 2 other (3.0%; 1 Danio rerio, 1 external Human Cell Atlas resource)
- **Cancer-focused studies:** 31 (47%)
- **Development-focused studies:** 22 (33.3%)
- **Multimodal studies:** 20 (30.3%)
